# Supplementary material for: Communities as ‘renewable energy’ for healthcare services? a multimethods study into the form, scale and role of voluntary support for community hospitals in England
Source: BMJ Open. 2019 Oct 7;9(10):e030243. doi: 10.1136/bmjopen-2019-030243 (PMC6797271; doi:10.1136/bmjopen-2019-030243)
Supplement: Supplementary data [file bmjopen-2019-030243supp001.pdf]

Communities as 'renewable energy' for health care services? A multi-methods study into the form, scale, and role of voluntary support for community hospitals in England

Angela Ellis Paine, Daiga Kamerāde, John Mohan and Deborah Davidson

Supplementary file: Interview topic guide summaries
Community Hospital research study: Profile, patient and carer experience, and community involvement and value

Staff

1) About you
    - Could you briefly tell me about your role in this community hospital?
    - How and why did you come to work at this hospital?
    - What is it like as a place to work?
2) Organisational background
    - Can you give me a basic introduction to this Community Hospital? (prompt for *history, services, staff, volunteers, funding, challenges and opportunities)*
3) Community context
    - Can you tell me a little bit about the local community: what is it like to live/work here?
4) Patient experience
    - How would you describe the patient experience at this community hospital?
    - Tell me about the main groups of patients who use this hospital?
    - What factors do you think are most significant in shaping patient experience?
    - What do you think of the facilities at this hospital? What kind of feedback do you get from the patients/carers about the facilities here?
    - What are the relationships like between patients and staff here?
    - What do you think about the level of information the hospitals provide the patients regarding their care, hospital facilities and wider services?
    - How involved do you think patients are in decisions about their care? In the hospital?
5) Community involvement
    - What are the different ways in which voluntary groups and the local community are involved in the Hospital, as providers, patients and residents? (probe around volunteering, governance, expert patients, services etc)
    - What does the community contribute to the hospital?
    - How is this organised and managed? (probe around recruitment, support, retention, use of external organisations)
    - How would the hospital be affected if the community did not get involved?
    - Do you think there is any relationship between community involvement and patient experience?
6) Value of community hospital to community
    - What does the community hospital contribute to the local community?
    - How would the community be affected if the community hospital wasn't here?
    - What does this community hospital mean to you?

Community stakeholders:

1) About you
   - Could you briefly tell me about you (and your role as [X])?
   - In what ways are you/your organisation involved in the community hospital?
2) Community hospital
   - How would you describe this community hospital?
3) Community context
   - Can you give me a basic introduction to the local community? What is it like to live/work here?
4) Patient experience
   - How would you describe the patient experience at this community hospital?
   - What factors do you think are most significant in shaping patient experience? (e.g. facilities, relationships, information, involvement)
5) Community involvement
   - What are the different ways in which voluntary groups and the local community are involved in the Hospital, as providers, patients and residents? (probe around volunteering, governance, expert patients, services etc)
   - What does the community contribute to the hospital?
   - How is this organised and managed? (probe around recruitment, support, retention, use of external organisations)
   - How would the hospital be affected if the community did not get involved?
   - Is there a connection between community involvement and patient experience?
6) Value of community hospital to community
   - What does the community hospital contribute to the local community?
   - How would the community be affected if the community hospital wasn't here?
   - What does the community hospital mean to you?

Volunteers

1) About you
   - Can you tell us a little bit about yourself, and about your role as a volunteer at [this] community hospital?
2) Getting involved
   - How did you come to be involved as a volunteer with the hospital? (prompt around being involved through another organisation, receiving services, or connections through family etc)
   - What motivated you to get involved?
3) Volunteering role
   - Can you tell us a bit more about what your role as a volunteer here? What does a typical day as a CH volunteer involve?
   - Has your role evolved over the course of the involvement in the CH?
   - Have you been able to do the kind of volunteer-roles that you hoped? If not, why not?
   - How frequently do you volunteer for the CH?
4) Volunteer support
   - What kind of support have you received as a volunteer at the hospital? (prompt for induction, training, supervision)

- How would you describe the relationship between volunteers and paid staff at the hospital?
- Overall, how helpful do you feel the support for volunteers is at the hospital? What is particularly good? What could be improved?

5) Volunteering outcomes
   - What difference do volunteers make to the community hospital as a whole?
   - What impact do volunteers have on the staff who work here?
   - What difference does the role of volunteers make to hospital patients, and carers? Can you give me any specific examples?
   - Is there anything that could be done differently to strengthen the outcomes for service users?
   - What difference has being involved as a volunteer at the CH made to you? (prompt for skills; friendships; enjoyment; employability)
   - Is there anything you don't like about being a volunteer here?
6) Value of community hospital to community
   - What does the community hospital contribute to the local community?
   - How would the community be affected if the community hospital wasn't here?
   - What does this community hospital mean to you?

Carer:

1) About you
   - Could you briefly tell me about you and your relationship to [patient]?
2) Services
   - What are the main services that you have had experience of through being a carer for a patient at this hospital?
3) Carer experience
   - Can you tell us about your experience of being a carer of a patient at [X] community hospital? (prompt for: care, support, treatment, facilities, relationships, involvement, feelings)
   - Was there anything that was particularly good about being a carer of a patient here?
   - Was there anything that was particularly difficult about being a carer of a patient here?
   - Was there anything that could have been done better to improve your experience of being a carer of a patient at [x] Community Hospital?
4) Community involvement
   - Have you been involved in the CH in any other way, such as being a patient, working here or being a volunteer?
   - How involved do you think the local community is in the hospital?
   - How would the hospital be affected if the community did not get involved?
5) Value of community hospital to community
   - What does the community hospital contribute to the local community?
   - How would the community be affected if the community hospital wasn't here?
   - What does the community hospital mean to you?

Patients (discovery interview)

Part A

1) We would like to hear about your experiences of this [name] community hospital. You may find it helpful to look at this picture and think about different elements of your experiences and factors that may have shaped them.

Prompts, if not covered in story, for: facilities, relationships, involvement in decisions, feelings

Part B

1) Was there anything that was particularly good about your experience of being a patient at [X] Community Hospital?
2) Was there anything that wasn't particularly good about your experience of being a patient at [x] Community Hospital?
3) What would have improved your experience of being a patient at [x] Community Hospital?
4) Have you ever been involved in the hospital in any way other than as a patient, such as being a member of staff, or a volunteer?
5) Finally a question we are asking everyone we speak with is "What does the Community Hospital mean to you?"
