# Supplementary material for: Communities as ‘renewable energy’ for healthcare services? a multimethods study into the form, scale and role of voluntary support for community hospitals in England
Source: BMJ Open. 2019 Oct 7;9(10):e030243. doi: 10.1136/bmjopen-2019-030243 (PMC6797271; doi:10.1136/bmjopen-2019-030243)
Supplement: Author's manuscript [file bmjopen-2019-030243.draft_revisions.pdf]

BMJ Open

BMJ Open is committed to open peer review. As part of this commitment we make the peer review history of every article we publish publicly available.

When an article is published we post the peer reviewers' comments and the authors' responses online. We also post the versions of the paper that were used during peer review. These are the versions that the peer review comments apply to.

The versions of the paper that follow are the versions that were submitted during the peer review process. They are not the versions of record or the final published versions. They should not be cited or distributed as the published version of this manuscript.

BMJ Open is an open access journal and the full, final, typeset and author-corrected version of record of the manuscript is available on our site with no access controls, subscription charges or pay-per-view fees (http://bmjopen.bmj.com).

If you have any questions on BMJ Open's open peer review process please email info.bmjopen@bmj.com

BMJ Open

Communities as 'renewable energy' for health care services? A multi-methods study into the form, scale and role of voluntary support for community hospitals in England

Journal:	*BMJ Open*
Manuscript ID	bmjopen-2019-030243
Article Type:	Research
Date Submitted by the Author:	05-Mar-2019
Complete List of Authors:	Ellis Paine, Angela; University of Birmingham, Third Sector Research Centre, School of Social Policy<br>Kamerāde, Daiga; University of Salford, School of Health and Society<br>Mohan, John; University of Birmingham, Third Sector Research Center (TSRC)<br>Davidson, Deborah; University of Birmingham, Health Services Management Centre
Keywords:	community hospital, volunteering, voluntary income, co-production, resources, change over time

SCHOLARONE™
Manuscripts

Communities as 'renewable energy' for health care services? A multi-methods study into the form, scale and role of voluntary support for community hospitals in England

Angela Ellis Paine, Daiga Kamerāde, John Mohan and Deborah Davidson

Corresponding author:

Angela Ellis Paine, University of Birmingham

Postal address: Third Sector Research Centre, Room 1151 Muirhead Tower, University of Birmingham, Edgbaston, Birmingham, B15 2TT

Email: a.ellispaine@bham.ac.uk

Phone: 07753747883

ORCID ID: 0000-0002-4385-5098

Co-authors:

Daiga Kamerāde, School of Health and Society, Salford University, Salford, UK. Email: D.Kamerade-Hanta@salford.ac.uk

John Mohan, Third Sector Research Centre, School of Social Policy, University of Birmingham, Birmingham, UK. Email: j.mohan@bham.ac.uk

Deborah Davidson, Health Services Management Centre, School of Social Policy, University of Birmingham, Birmingham, UK. Email: d.c.davidson@bham.ac.uk

Article word count: 3,926

For peer review only

Communities as 'renewable energy' for health care services? A multi-methods study into the form, scale and role of voluntary support for community hospitals in England

KEY WORDS

Community hospitals, volunteering, voluntary income, resources, co-production

ABSTRACT

Objective

To examine the forms and levels of community and voluntary support for community hospitals in England.

Design

A multi-methods study. Quantitative analysis of Charity Commission data on volunteering, voluntary income and expenditure for charities supporting community hospitals. Nine qualitative case studies of community hospitals and their surrounding communities, including interviews and focus groups.

Setting

Community hospitals in England and their surrounding communities.

Participants

Charity Commission data for 245 community hospital Leagues of Friends. Interviews with staff (89), patients (60), carers (28), volunteers (35), community representatives (20), managers and commissioners (9). Focus groups with multi-disciplinary teams (8 groups across nine sites, involving 43 respondents), volunteers (6 groups, 33 respondents) and community stakeholders (8 groups, 54 respondents).

Results

Communities support community hospitals through: *human resources* (average = 24 volunteers a year per hospital); *financial resources* (median voluntary income = £15,632); *practical resources* through services and activities provided by voluntary and community groups; and *intellectual resources* (e.g. consultation and coproduction). Communities provide valuable supplementary resources to the NHS, enhancing community hospital services, patient experience, staff morale and volunteer well-being. Such resources, however, vary in level and form from hospital to hospital and over time: voluntary income is on the decline, as is membership of League of Friends, and it can be hard to recruit regular, active volunteers.

For peer review only

Conclusions

Communities can be a significant resource for health care services, in ways which can enhance patient experience and service quality. Harnessing that resource, however, is not straight forward and there is a perception that it might be becoming more difficult questioning the extent to which that resource can be considered to be 'renewable energy'.

ARTICLE SUMMARY: STRENGTHS AND LIMITATIONS

- A robust multi-methods study allowing us to understand the breadth, depth and variability of community support for community hospitals.
- Original insights and data on the levels and forms of voluntary support for community hospitals, which had previously been almost non-existent.
- Limited by the availability of secondary data for the detailed analysis of expenditure from charitable funds.
- Limitations associated with a majority of our interviewees having had some connection to the community hospital: few who had no connection at all.

For peer review only

INTRODUCTION

A growing recognition of a need to find new ways to deliver public services, has contributed to an increasing emphasis on involving communities and individual volunteers in the NHS. The Five Year Forward View [1] identifies communities and patients as 'renewable energy', suggesting they are seen as a resource that can be naturally replenished to support the NHS. The NHS Long Term Plan [2] commits to supporting the *HelpForce* [3] initiative, which aims to double the number of volunteers in the NHS. Some have gone as far as to suggest the NHS would collapse without the support of volunteers [4]. Such claims, however, are often made with limited evidence. We know relatively little about how extensive community involvement is within the NHS, what form it takes, how it varies, or to what extent this 'energy' can be considered 'renewable'.

Community hospitals are a long standing part of the healthcare landscape. In 2015, there were 296 community hospitals with beds in England, with a particular concentration in rural areas, providing a valuable, but varied, mix of intermediate care services [5]. They have a history of community involvement, often having started through local people raising funds [6]. After being transferred to the NHS, the traditions of voluntary support were maintained, particularly through the formation of Leagues of Friends. While historical accounts have highlighted substantial local commitment to individual community hospitals [7, 8, 9] contemporary empirical analysis has been lacking. We are aware of only one academic study of Hospital Leagues of Friends in the UK, published in 1960 [10].

Existing national survey evidence provides only a very general insight. Naylor *et al.* [11] estimate that approximately 2.9 million people in England volunteer for health-related organisations and causes, but cannot break these figures down any further. Studies of voluntary income for the NHS or other specific subsectors of healthcare [12] have focused on relatively large organisations or looked at data across authorities meaning that levels of support for individual institutions cannot be identified [13]. Galea *et al*'s [14] study of volunteering in NHS acute Trusts in England found that on average they involve 471 volunteers each, a total of 78,000 people who together contributed 13 million hours per year. The study highlighted variations in between Trusts, but as a cross-sectional study did

For peer review only

not offer insights into changes over time and focused only on one form of voluntary support in one particular setting.

A small number of studies have considered the outcomes of certain forms of community engagement for hospitals and for the wider healthcare system. There is some evidence [15, 16] that volunteers can have a positive impact on healthcare systems, through improving patient experience, strengthening the relationships between services and community, improving public health and supporting integrated care. Such evidence, however, is limited.

This paper fills some of these evidence gaps, drawing on a multimethod study investigating the different ways in which communities are involved in and support their community hospitals. We discuss the extent to which community support for community hospitals may be considered a form of 'renewable energy', which may become important as other resources are stretched increasingly thinly.

METHODS

We draw on evidence from a large national multimethod study exploring the profile, characteristics, patient experience, community engagement and value of community hospitals [17]. One aim of this wider study was to address the question: 'what do communities do to support their community hospitals?'

To examine levels of volunteering and financial support for community hospitals, a dataset was compiled from Charity Commission records. The sample for this study was 245 community-based charities (Leagues of Friends and other similar charities) in England that directly support their local community hospitals and for which financial information was available for at least one year between 1995 and 2014. The Charity Commission records included data on the finances and volunteer numbers for each charity. We conducted a more detailed analysis of income sources and types of expenditure using the annual financial accounts of those charities whose income or expenditure exceeds the threshold (£25,000 per year) above which financial accounts are made available on the Charity Commission website. This gave data covering 358 separate financial years; the average number of charities with detailed accounts was 60 per financial year. At the time of

For peer review only

conducting analysis, the latest detailed financial information available was for the financial year 2013. The number of accounts available varies from year to year because an individual charity may or may not exceed the £25,000 threshold, depending on fluctuations in its finances. All financial information in this paper is presented at constant 2014 prices. We undertook descriptive analysis of variations and a fixed-effects regression analysis to explore changes over time.

To explore the experiences and perceived outcomes of community support, nine qualitative case studies were undertaken. The case studies were selected from a comprehensive database of community hospitals in England, to reflect diversity in terms of: location (north / south; rural/urban; deprivation), size (number of beds; range of services), models of ownership and provision (public/voluntary); and levels of voluntary income [18].

Each case study involved: scoping; local reference groups (staff and community members brought together to inform the study and reflect on emerging findings); semi-structured interviews with: Trust managers and commissioners (9, across the 9 cases), staff (89), volunteers (35), community stakeholders (20) and carers (28); discovery interviews with patients (60); focus groups with multi-disciplinary teams (8, across the nine sites, involving 43 respondents), volunteers (6 groups, 33 respondents) and community stakeholders (8 groups, 54 respondents).

Interviews and focus groups were recorded and transcribed verbatim before being imported into NVivo11 software, and analysed thematically. A coding frame was developed collaboratively by the qualitative research team, mixing inductive and deductive codes. After an initial testing of the coding frame, codes were refined, reordered and grouped, before the final version was applied across the whole dataset.

Data convergence took place throughout the analysis period, with regular whole-team meetings convened to discuss emerging findings from across the different stands of analysis.

Patient and public involvement

For peer review only

Key stakeholders - including members of the Community Hospitals Association, community hospital staff, patients, carers and the public - were involved in the design and delivery of this study, including through a national Steering Group and Local Reference Groups. Emerging findings were discussed with patient, community and staff groups at national and local level through a range of forums including Local Reference Group meetings, Annual Learning Events, and conferences.

RESULTS

The study identified four, inter-related, types of resource that communities provide for their hospitals, often channelled through the League of Friends.

Human resource: giving time through volunteering in community hospitals

Analysis of Charity Commission (CC) records found that Community Hospital Leagues of Friends on average involved 24 volunteers (range 2 to 162): an estimated total of 5,880 volunteers across the 245 community hospitals in the sample. This equates to between 1.4 and 2.5 full time equivalent personnel. If expressed in terms of the national minimum wage, the volunteer labour provided by Leagues of Friend equates to a financial input of £15,600–£28,500 per community hospital, or £3.8million to £6.9 million across England. These figures are likely to be underestimates: they exclude trustees and those who volunteer within community hospitals but outside the League of Friends; and the calculations used only national averages for volunteer hours and the minimum national hourly wages.

Our case studies suggest that the volunteers tended to be retired, female, white and middle class. Social capital plays an important role in maintaining community support: most volunteers become involved through word of mouth, thus limiting the potential pool of new volunteers. Examples of active volunteer recruitment campaigns were limited, and concerns were raised about the challenge of involving new people. While there were generally high levels of latent support for community hospitals, particularly demonstrable at times of threat to services, a common perception was that it was difficult to convert this into active, regular, volunteer engagement:

For peer review only

'I think that a lot of people would go up in arms if it was closed but whether they'd be willing to do anything about it to help I don't know. There seems to be a little core of people that would but I'm not sure that that spreads right out across the community'
CH9, Carer

Not all volunteering resources are renewable. In one community hospital the League of Friends had ceased to operate as previous volunteers retired and they had been unable to recruit new members to take over. In another, however, volunteer involvement had been reinvigorated through the recruitment of a new League of Friends chair and active support from the hospital matron.

We identified seven categories of volunteer roles: campaigning and advocacy; governance, leadership and management; fundraising; administrative assistance; service delivery; facilities; and patient well-being. There was a clear consensus that volunteers could not get involved in medical or personal care or access confidential data and that they were there to complement, rather than supplement, paid staff. There was far less consensus on the actual roles that they could undertake, with considerable variations found across the sites.

Health and safety regulations and concerns about confidentiality were the most commonly cited restrictions on volunteer involvement. In some, they were seen as reasons not to involve volunteers, in others they were seen to represent a set of training requirements and risk management. Volunteer involvement was further limited by perceptions of key actors within the hospitals, including concerns about: the capacity of patients to engage with volunteers; the views of carers/families towards volunteers; capacity of staff to facilitate volunteering; and the willingness of people to volunteer. Some respondents argued that such concerns were putting *'barriers up where they don't exist'* and as such hospitals were not always making the most of *'untapped resource'* available to them.

Financial resources: giving money through fundraising for community hospitals

Leagues of Friends provide substantial financial resource. In 2014, on average, community hospital League of Friends generated financial resources worth £45,387 (Figure 1). This

For peer review only

figure is influenced by a small number of very large outliers: the median income was £15,632. In addition, some community hospitals benefit from income generated through donations made directly to NHS Trusts: these funds are not counted in the above figures (our quantitative data is confined to League of Friends) but were reported to be significant in three case studies.

The community hospital Leagues of Friends were both relatively large and relatively stable entities within their local charitable landscapes: 40% were among the largest 100 charities in their local authorities in terms of income.

Charities generate financial resources for community hospitals through various means. Figure 2 presents an overview of income sources, derived from charity accounts from Leagues of Friends with an income or expenditure greater than £25,000 in any given year. Over a five-year period from 2008-2013, approximately three-quarters (77%) of Leagues of Friends' income was 'voluntary income', that is, an income generated through legacies (particularly significant), gifts, donations, grants, membership subscriptions and sponsorships. One-sixth (15%) of the total income was from activities for generating funds, such as jumble sales, lotteries, and charity shops. Less than one-tenth was raised through 'charitable activities', such as trading goods and services.

Figure 3 shows how average levels of the total income and expenditure have varied over time. The trend in income is downwards: League of Friends' incomes have been declining since 1995. The decline was most pronounced in 2011 and 2012, showing some signs of recovery in 2013 and 2014. Fixed-effects regression analysis, entering a constant term and year as the only predictors, in order to compare income with that of a base category (income in 1995), indicates that these temporal changes were statistically significant. The average and median income that Leagues of Friends have received yearly was on the rise until the mid-2000s but since then has declined; the decline in income in general has been statistically significant ($b= -901.29$, $p < .01$). Since 1995 the charitable income of Leagues of Friends has declined on average by approximately £901 a year.

For peer review only

Notwithstanding this finding, there was little evidence amongst our case studies of concern over declining levels of income. Indeed, some had limited their fundraising activities, either through recognising a potential crowding out of other local charities or due to uncertainty over the future of the hospital. However, some questioned the future sustainability of income levels, and raised concerns about declining membership levels, which was seen as a harbinger of more general reduced community support.

Expenditure had also declined, but less sharply than income (see Figure 3). Since 1995 community hospital Leagues of Friends have, in most years, received more than they have spent. The exceptions from this trend are years 1997 and 2011 when an average Friends group spent more than it received. Overall, within the dataset that we have, it appears that in 2011 their funding position was at its weakest since 1995 (figure 3).

These financial resources are used to provide various amenities for community hospitals: patient comforts (found across all case studies); staff development; equipment; buildings; staff time (found in a small number of cases). There was some uncertainty about where the boundary between voluntary and statutory support should be drawn. Some cases adopted a clear demarcation avoiding anything they felt to be a statutory responsibility (e.g. buildings, equipment, staffing), others did not. One League of Friends, for example, was paying for a chaplain, a gardener and had temporarily funded the extension of the opening hours of the Minor Injuries Unit. Overall, it was felt, the boundaries were becoming increasingly blurred:

> *'We are supposed to help the hospital for extras. This was how it started off. It has got to be very much more mainstream now because of lack of funds' CH9, Volunteer*

Practical resources: Providing services through voluntary organisations

Various national and local voluntary organisations also contributed to community hospitals, providing some direct form of service to patients getting into hospital, during their stay, or on discharge. They also provided support for families and carers of hospital patients, or more general support for the hospital through, for example, running fundraising events. Many of these services were free to the hospitals, some were paid for; some were delivered by paid staff employed by the voluntary organisations and others by volunteers. In some

For peer review only

cases voluntary groups, or members of staff from within them, were co-located within the hospital, providing important mutual benefits.

The interaction of community hospitals with such voluntary organisations varied. The general perception was that more could be done to strengthen the links between the community hospital and local voluntary and community groups. A lack of knowledge or capacity amongst hospital staff to identify and build relationships with relevant organisations was identified as a barrier.

Intellectual resources: giving voice through information, consultation and co-production

Leagues of Friends in particular, but also other community groups and individuals, played a role in supporting the hospital through representing the hospital in the community and/or vice versa. This happened at different levels.

At the most basic level, an important role for the League of Friends was to provide a communication channel between the hospital and its community. The Leagues of Friends helped raise the profile of the hospital and provide feedback on services. Many League of Friends members were well networked within their local communities often being members of multiple local groups and organisations, facilitating this role.

For peer review only

Leagues of Friends had come, by design or default, to represent the 'voice of the community' in various consultation processes concerning the future of the hospital/ services. This wasn't always a role they had anticipated, or one which all had the capacity to fulfil. Considerable frustration was expressed about such consultations, focusing on: poor processes, lack of timely communication, uneven engagement, and an apparent inability to influence outcomes. Consultations were often dismissed by community members as tokenistic. Commissioners recognised the importance of consultation, suggesting communities would react '*more reasonably*' to CCG-led changes if they had been involved in the decisions. Some commissioners, however, also expressed their own frustrations about the consultation processes, suggesting for example that:

> *'however much you do it's never enough and some people will always feel that they've in some way been excluded.'* (CH8&9, Commissioner)

Examples of deeper, sustained community involvement in the ongoing coproduction of community hospitals services were limited. There was frustration that opportunities were not being taken to maximise the potential for co-production and to draw upon the expertise and energy of the community:

> *'There is no local input at all and I think there should be. You call it a local reference group: I would love to have a small local reference group and that would at least – not run the hospital – but at least be able to have a say and it could be listened to' (CH2, Volunteer)*

Amongst the nine case studies, however, there were two significant exceptions. In one, following a decision by the NHS to close the hospital, the local community established a charity and ran a successful fundraising campaign to re-open the hospital. The charity now owns and runs the hospital, with services provided by the NHS, and has established a range of other health and social care services on the same site. In another, again following (perceived) threats to the future of the hospital, the community formed a local action group which has become a key player in a now well established local health forum which brings

For peer review only

together statutory health and social care providers together with the local community to co-design and co-deliver services on an ongoing basis.

Making a difference

Distilling the combined impact of these (often taken for granted) resources is difficult. Some suggested that community involvement was '*key to the community hospital'*: it was what makes a hospital a '*community* hospital'; and that community hospitals would be a '*totally different place'* without it. In one, however, it was suggested that if the community didn't get involved no-one would really notice, although *'some of the trimmings might disappear round the edge'.* The study identified five sets of outcomes:

- Enhancing community hospital utilisation and resilience: There were hospitals that had been established through considerable community effort, which had been sustained post-nationalisation, and had been kept open despite considerable pressures to rationalise. Community engagement, it was suggested, may have been an important factor in the apparent resilience of community hospitals which the stability (at least until recently) in their numbers suggests [19]. Community involvement had also contributed to service utilisation in some hospitals: through raising awareness of facilities available either directly through specific campaigns or indirectly through fundraising activities. However, a tendency towards resisting change was seen by some to be a downside of community engagement, potentially preventing improvements to service delivery.

- Contributing to patient and carer experience: Enhancements hospital buildings and equipment, resulting from voluntary efforts, were agreed to contribute to patient experience, giving them the *'extra bits which the hospital won't or can't afford',* and contributing to the *'happy atmosphere'* found in most of the case study community hospitals. It was suggested that some patients found it easier to talk to volunteers ('*someone out of uniform')* than to staff. Volunteers, it was suggested, could help to tackle isolation, loneliness and boredom, and more generally to '*lift peoples' spirits'* and '*making them happy*'. Community engagement was also thought to be beneficial at the point of discharge, easing the transition from hospital to home.

- Boosting staff morale: Community involvement also had an effect on staff. The perception of being supported and valued by the community contributed to an identified distinction between the experience of working in a community hospital and an acute hospital, could help boost staff morale, and build a sense of loyalty to the hospital. On the other hand, some staff reported having to invest valuable time in facilitating community engagement, 'pick up' tasks when volunteers had not turned up or had not done a job effectively, and deal with the expectations of community members that arose from their support of the hospital (e.g. an entitlement to access beds).

- Enhancing volunteer well-being: Engaging with the hospital also had outcomes for the volunteers themselves. Volunteering in the hospital/League of Friends provided structure for some, and acted as a replacement for paid work, or as an alternative to it. The social interaction, physical and mental activity associated with volunteering was also important for some, particularly older, respondents. However, for some, volunteering encroached on personal and family time and could be experienced as stressful and tiring, particularly when it proved difficult to recruit enough helpers.

- Community well-being: the activities involved in supporting community hospitals were building, and often reliant upon social interaction, networks and trust. Fundraising events in particular were highlighted as important functions in the social calendar of some communities, bringing people together and tackling social isolation. This was seen as particularly important for older people.

DISCUSSION

Community hospitals are generally well supported by their local communities. Many benefit from considerable input of resources in terms of time, money, service and intelligence. Together through this voluntary 'energy', communities provide significant additional resources to their community hospitals. As previous studies have suggested, it can positive affect patient experience [20, 21, 22] and service quality by adding capacity, enhancing facilities, and boosting staff morale [23].

For peer review only

However, levels of voluntary income have been declining (in a study of charity finances, Clifford [24] likewise found that Leagues of Friends were one of the groups of charities that had experienced a decline in income in recent years). Membership has also declined, and it was increasingly hard to recruit active regular volunteers. While the study identified widespread latent support for community hospitals, regular, ongoing, active involvement was often limited to a relatively small group of volunteers. Getting new and particularly younger people to actively engage in community hospitals on an ongoing basis - beyond moments of crisis - was a challenge. The variance in levels of community support, between and within communities and over time, echoes studies of voluntary support in other areas of healthcare [25, 26] as well as in other sectors [27, 28]. These findings question the extent to which voluntary and community support can be always be considered a source of 'renewable energy'.

While we agree with Munoz *et al.*'s [29] conclusion that *'harnessing more local volunteers […] is more complex than governments assume'* (p. 221), we found limited evidence of extensive efforts being made to do so. Several limits to the involvement of volunteers and voluntary groups were identified, alongside a general lack of 'investment' in their engagement beyond that provided by League of Friends or individual staff. Converting the extensive (often passive) support found across communities into more regular, active engagement, beyond moments of crisis, requires investment of time, enthusiasm and inevitably money. Just as harnessing other sources of renewable energy (e.g. wind, sun) requires investment in the appropriate infrastructure, so too – we suggest - does voluntary and community support.

This study has provided original insights into voluntary support for community hospitals, demonstrating variations in its contribution, its reliance on a relatively small number of committed individuals, and raises questions as to the extent to which it can be considered 'renewable'. This provides important lessons for policies predicated on the expansion of voluntary and community engagement in health care.

For peer review only

Study limitations include limits to the secondary data available for the detailed analysis of expenditure from charitable funds, and the concentration amongst our interviewees on respondents with some connection to the community hospital.

Future research priorities include longitudinal research to explore changes in the levels and forms of voluntary support and comparative studies of voluntary support in different health care settings.

ETHICAL APROVAL

The Wales Research Ethics Committee 6 reviewed this research study reference: 16/WA/0021, on the 24th February, 2016, and provided a favourable ethical opinion. All research participants gave consent to be involved in the study.

ACKNOWLEDGEMENTS

We would like to thank all our study respondents who contributed to this study through participating in interviews, focus groups, site visits and responding to requests for information. Particular thanks go to matrons/ward sisters, community hospital clinicians and League of Friends members who coordinated research activities for us, organized Local Reference Groups, and participated in learning events. We would also like to thank our funders, the National Institute for Health Research, and fellow researchers who contributed to the study as highlighted in the 'author contributions' section.

AUTHOR CONTRIBUTIONS

All authors were involved in the writing of the article, with Angela taking the lead. Angela and Deborah led on the qualitative aspects of the study, John and Daiga on the quantitative. We would also like to acknowledge the contributions of other members of the study team who were involved in study design and data collection but were not directly involved in authoring the article: Nick Le Meseurier, Helen Tucker, John Glasby, Iestyn Williams, David Seamark, Tessa Crilly, John Crilly and Jan Marriot.

FUNDING

This work was supported by The National Institute for Health Research - Health Services and Delivery Research: New Research on Community Hospitals, grant number 12/177/13.

For peer review only

COMPETING INTERESTS

None

DATA SHARING

The quantitative datasets for the study are available from the UK Data Archive: Alcock, Peter and Mohan, John (2013). *Third Sector Research Centre research data collection.* [Data Collection]. Colchester, Essex: Economic and Social Research Council. 10.5255/UKDA-SN-850933

REFERENCES

1 NHS England *Five Year Forward View* London: HMSO 2014.

2 NHS England *The NHS Long Term Plan* London: HMSO 2019.

3 https://www.helpforce.community/ accessed on 05.01.19

4 Dudman J. ''The NHS would collapse without them': the growing role of volunteers' *The Guardian, 04.07.18*

5 Davidson D, Ellis Paine A, Glasby J et al Analysis of the profile, characteristics, patient experience and community value of community hospitals: a multimethod study. *Health Service Delivery Research 2019* 7(1).

6 Hall J. From cottage to community hospitals: Watlington Cottage Hospital and its regional context, 1874-2009. *Local Population Studies* 2012 88(1): 33-49.

7 Hall J. 2012 op. cit.

8 Sutton C. Axminster Hospital 1886-1986. 1986.

9 Emrys Roberts M. Cottage Hospitals 1859-1990: Arrival, survival and revival. Dorset: Turn Publications, 1991.

10 Cooney E. The Leagues of Hospital Friends. *Public Administration* 1960, 38:263-272.

11 Naylor C, Mundle C, Weaks L, Buck D. Volunteering in health and care: Securing a sustainable future. Kings Fund: London 2013.

12 Mocroft I, Pharoah C. Coming full circle: charitable funds in London's health. London: King's Fund 2001.

13 Lattimer M. The gift of health. London: Directory of Social Change 1996.

14 Galea A, Naylor C, Buck D, Weaks L. Volunteering in acute trusts in England: understanding the scale and impact. London: The Kings Fund 2013.

For peer review only

15 Naylor et al. 2013 op. cit.

16 Mundle C, Naylor C, Buck D. Volunteering in health and care in England. London: The Kings Fund 2012.

17 Davidson et al. 2019. op. cit.

18 See Davidson et al. 2019. op. cit. for further details

19 Davidson et al. 2019. op. cit.

20 Hotchkiss R, Unruh L, Fottler M. The role, measurement and impact of volunteerism in hospitals. *Nonprofit and Voluntary Sector Management* 2014 43(6):1111-1128.

21 Naylor et al. 2013 op. cit.

22 Kang R, Hasnain-Wynia R, Hood M. Hospital commitment to community orientation with its association with quality of care and patient experience. *Journal of Healthcare Management* 2013 58(4): 277-288.

23 Mundle et al. 2012. op. cit.

24 Clifford D. Charitable organisations, the great recession and the age of austerity: longitudinal evidence for England and Wales. *Journal of Social Policy* 2017 46(1): 1-30.

25 Munoz S-A, Farmer J, Warburton J, Hall J. Involving older people in service co-production: is there an untapped pool of potential participants? *Journal of Rural Studies* 2014 34(3):212-222.

26 Galea et al. 2013. op cited

27 Mohan J, Bulloch C. *Is there a British 'civic core'? Evidence from the Citizenship Survey.* TSRC Working Paper No. 73; Birmingham: Third Sector Research Centre 2012.

28 Wilson J, and Musick M. The Contribution of Social Resources to Volunteering. *Social Science Quarterly* 1998 79(4): 799–814.

29 Munoz et al. 2014. op. cit.

LIST OF FIGURES

Figure 1: League of Friends income distribution in 2014

Figure 2: Community Hospital Leagues of Friends' income sources, by year

Figure 3: Leagues of Friends income and expenditure over time

For peer review only

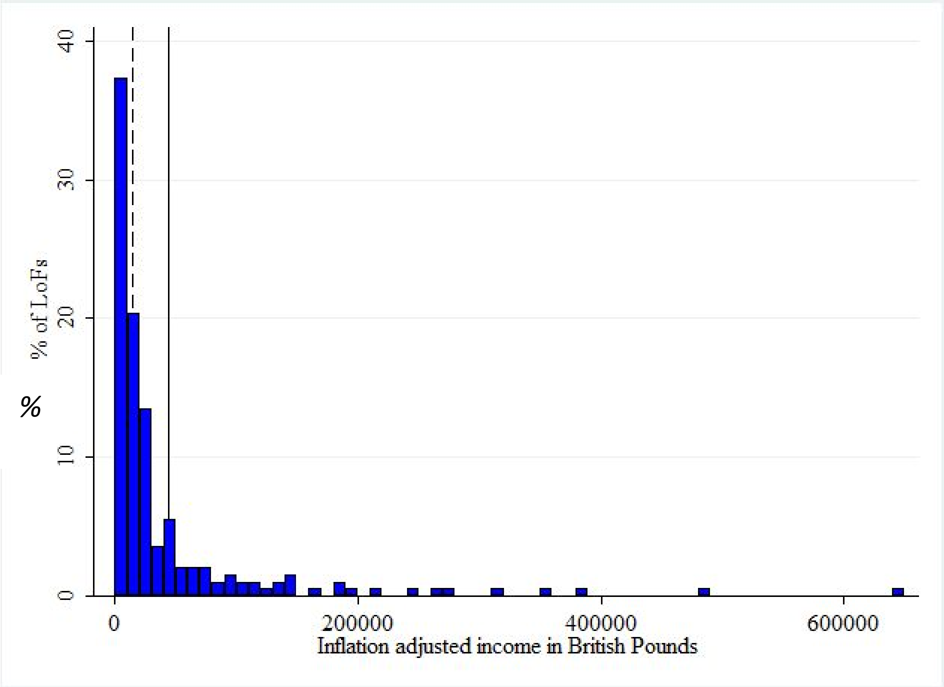

Figure 1: League of Friends income distribution in 2014

Notes: solid line= mean (£45,387); dashed line= median £15,632)

Reprinted with permission from Davidson D, Paine AE, Glasby J, Williams I, Tucker H, Crilly T, et al. Analysis of the profile, characteristics, patient experience and community value of community hospitals: a multimethod study. Health Serv Deliv Res 2019;7(1)

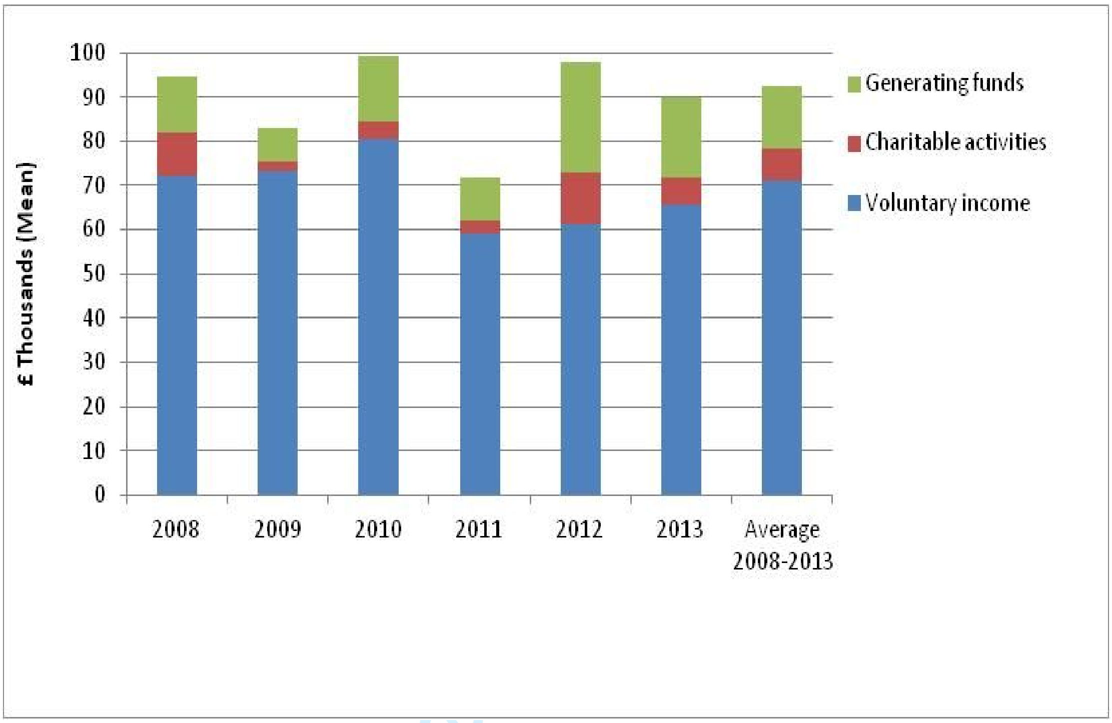

Figure 2: Community Hospital Leagues of Friends' income sources, by year

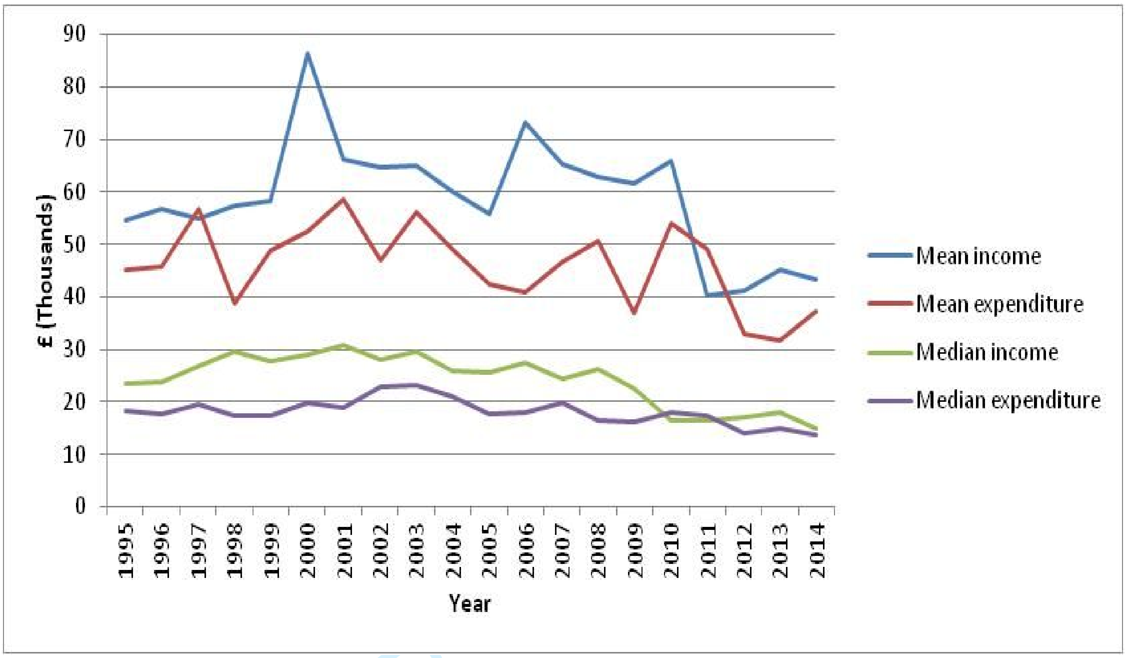

Figure 3: League of Friends income and expenditure over time

For peer review only

BMJ Open

Communities as 'renewable energy' for health care services? A multi-methods study into the form, scale and role of voluntary support for community hospitals in England

Journal:	*BMJ Open*
Manuscript ID	bmjopen-2019-030243.R1
Article Type:	Original research
Date Submitted by the Author:	15-Jul-2019
Complete List of Authors:	Ellis Paine, Angela; University of Birmingham, Third Sector Research Centre, School of Social Policy<br>Kamerāde, Daiga; University of Salford, School of Health and Society<br>Mohan, John; University of Birmingham, Third Sector Research Center (TSRC)<br>Davidson, Deborah; University of Birmingham, Health Services Management Centre
**Primary Subject Heading**:	Health services research
Secondary Subject Heading:	Qualitative research, Health policy
Keywords:	community hospital, volunteering, voluntary income, co-production, change over time, sustainability

SCHOLARONE™
Manuscripts

Communities as 'renewable energy' for health care services? A multi-methods study into the form, scale, and role of voluntary support for community hospitals in England

Angela Ellis Paine, Daiga Kamerāde, John Mohan and Deborah Davidson

Corresponding author:

Angela Ellis Paine, University of Birmingham

Postal address: Third Sector Research Centre, Room 1151 Muirhead Tower, University of Birmingham, Edgbaston, Birmingham, B15 2TT

Email: a.ellispaine@bham.ac.uk

Phone: 07753747883

ORCID ID: 0000-0002-4385-5098

Co-authors:

Daiga Kamerāde, School of Health and Society, Salford University, Salford, UK. Email: D.Kamerade-Hanta@salford.ac.uk

John Mohan, Third Sector Research Centre, School of Social Policy, University of Birmingham, Birmingham, UK. Email: j.mohan@bham.ac.uk

Deborah Davidson, Health Services Management Centre, School of Social Policy, University of Birmingham, Birmingham, UK. Email: d.c.davidson@bham.ac.uk

Article word count: 4,274

For peer review only

Communities as 'renewable energy' for health care services? A multi-methods study into the form, scale and role of voluntary support for community hospitals in England

KEY WORDS

Community hospitals, volunteering, voluntary income, co-production, sustainability

ABSTRACT

Objective

To examine the forms, scale and role of community and voluntary support for community hospitals in England.

Design

A multi-methods study. Quantitative analysis of Charity Commission data on levels of volunteering and voluntary income for charities supporting community hospitals. Nine qualitative case studies of community hospitals and their surrounding communities, including interviews and focus groups.

Setting

Community hospitals in England and their surrounding communities.

Participants

Charity Commission data for 245 community hospital Leagues of Friends. Interviews with staff (89), patients (60), carers (28), volunteers (35), community representatives (20), managers and commissioners (9). Focus groups with multi-disciplinary teams (8 groups across nine sites, involving 43 respondents), volunteers (6 groups, 33 respondents) and community stakeholders (8 groups, 54 respondents).

Results

Communities support community hospitals through: *human resources* (average = 24 volunteers a year per hospital); *financial resources* (median voluntary income = £15,632); *practical resources* through services and activities provided by voluntary and community groups; and *intellectual resources* (e.g. consultation and coproduction). Communities provide valuable supplementary resources to the NHS, enhancing community hospital services, patient experience, staff morale and volunteer well-being. Such resources, however, vary in level and form from hospital to hospital and over time: voluntary income is

For peer review only

on the decline, as is membership of League of Friends, and it can be hard to recruit regular, active volunteers.

Conclusions

Communities can be a significant resource for health care services, in ways which can enhance patient experience and service quality. Harnessing that resource, however, is not straight forward and there is a perception that it might be becoming more difficult questioning the extent to which it can be considered sustainable or 'renewable'.

ARTICLE SUMMARY: STRENGTHS AND LIMITATIONS

- A robust multi-methods study allowing us to understand the breadth, depth and variability of community support for community hospitals.
- Original insights and data on the levels and forms of voluntary support for community hospitals, which had previously been almost non-existent.
- Limited by the availability of secondary data for the detailed analysis of levels of volunteering (particularly outside of Leagues of Friends) and expenditure from charitable funds.
- Limited by its scope to consider the wider system within which community hospitals are embedded.

For peer review only

INTRODUCTION

A growing recognition of a need to find new ways to deliver public services has contributed to an increasing emphasis on involving communities and individual volunteers in the NHS. NHS England's Five Year Forward View, which in 2014 set out a vision for the future of the NHS, identified communities and patients as 'renewable energy'.[1] This suggests they are seen as a sustainable resource that can be naturally replenished to support the NHS. More recently, NHS England's 'NHS Long Term Plan' has committed to supporting the aim of doubling the number of volunteers in the NHS.[2] Some have gone as far as to suggest the NHS would collapse without the support of volunteers.[3] More broadly, Crisp identifies voluntary and community organisations as part of the 'informal system of care', which, if strengthened, could relieve pressure on health and social care services, contributing to their sustainability.[4] Such claims, however, are often made with limited evidence. We know little about how extensive community involvement is within the NHS, what form it takes, how it varies, or to what extent this 'energy' can be considered sustainable or 'renewable'.

Community hospitals are a long standing part of the healthcare landscape. In 2015, there were 296 community hospitals in England. Community hospitals are small (typically less than 30 beds), predominantly rural, traditionally GP-led, and provide a varied mix of intermediate care services.[5] They often originated as cottage hospitals through local voluntary initiative.[6] Under NHS control, the traditions of voluntary support were maintained, particularly through the formation of Leagues of Friends – independent charities set up to raise funds and mobilise support. Historical accounts have highlighted substantial local commitment to individual community hospitals[6-8], but we are aware of only one academic study of Hospital Leagues of Friends in the UK, published in 1960.[9]

Existing national survey evidence provides only a very general insight into voluntary action in the NHS. Naylor *et al.* estimate that approximately 2.9 million people in England volunteer for health-related organisations and causes, but cannot break this down by specific settings.[10] Studies of voluntary income in healthcare have focused on relatively large organisations[11] or looked at data across authorities.[12] Galea *et al*'s study of volunteering in NHS Acute (secondary care) Trusts in England found that on average they

For peer review only - http://bmjopen.bmj.com/site/about/guidelines.xhtml

involve 471 volunteers each.[13] The study highlighted variations between Trusts, but focused only on one form of voluntary support in one particular setting.

A small number of studies have considered the outcomes of certain forms of community engagement for hospitals and for the wider healthcare system. There is some evidence that volunteers can have a positive impact on healthcare, through improving patient experience, strengthening the relationships between services and community, improving public health and supporting integrated care.[10,14] Such evidence, however, is limited.

To investigate how communities support their community hospitals we draw on a multi-methods study. The study offers original evidence on the scale, form and role of voluntary support for community hospitals, and variations over time. Given current and likely future pressures on resources within the NHS, we reflect upon the extent to which voluntary support may be considered a sustainable form of 'renewable energy' offering important additional resources for hospitals, patients and communities.

METHODS

We draw on evidence from a large multi-methods study exploring the profile, characteristics, patient experience, community engagement and value of community hospitals.[5] One aim of this wider study – and of this paper – was to address the question: 'what do communities do to support their community hospitals?' More specifically, the intention is to explore the scale, forms, role and character of voluntary support to community hospitals and how these have changed over time. Developing a complete understanding of voluntary support required a mix of both quantitative (how much) and qualitative (how and why) data. A convergent design was employed, with the qualitative and quantitative elements conducted broadly in parallel and given equal status. Different researchers were involved in the two elements. The research followed good practice guidelines on mixed methods studies.[15, 16]

The quantitative element involved examining the scale of volunteering and financial support for community hospitals through compiling and analysing a dataset of records held by the Charity Commission (the charity regulator in England and Wales). The sample for this study

For peer review only

was 245 Leagues of Friends (and other similar charities) in England that directly support community hospitals and for which financial information was available for at least one year between 1995 and 2014. The Charity Commission records included data on the finances and volunteer numbers for each charity.

We conducted a more detailed analysis of income sources and types of expenditure using data captured from the annual financial accounts of those charities whose income or expenditure exceeds the threshold (£25,000 per year) above which financial accounts are made available on the Charity Commission website. This gave data covering 358 sets of accounts across six financial years; the average number of charities with detailed accounts was 60 per financial year. At the time of conducting analysis, the latest detailed financial information available was for the financial year 2013. The number of accounts available varies from year to year because an individual charity may or may not exceed the £25,000 threshold, depending on fluctuations in its finances. All financial figures were adjusted for inflation using the Office for National Statistics Retail Prices annual index; therefore all financial information in this paper is presented in 2014 prices.

We undertook descriptive analysis of variations and used a fixed effects regression model[17] to examine how within-hospital charity changes in time (years) are linked to changes in the annual income within each individual charity over time, while eliminating unobserved heterogeneity – confounding effects from time-constant variables. The year variable examines any variation in the outcome that happen over time.

The qualitative element involved adopting a case study approach to explore the form, role, experiences and perceived outcomes of community support, reported here using the SRQR guidelines.[18] Nine case studies were selected from a database of community hospitals in England, reflecting diversity in terms of: location, size, models of ownership and provision, and levels of voluntary income (from well below average to well above).

Each case study involved: scoping; a local reference group (staff and community members brought together to inform the study and reflect on emerging findings); semi-structured interviews with: Trust managers and commissioners (9, across the 9 cases), staff (89),

For peer review only

volunteers (35), community stakeholders (20) and carers (28); discovery interviews with patients (60); focus groups with multi-disciplinary teams (8, across the 9 sites, involving 43 respondents), volunteers (6 groups, 33 respondents) and community stakeholders (8 groups, 54 respondents). We used purposive sampling, informed by our scoping visits, local reference groups and snowball techniques, with respondents selected due to their involvement in or knowledge of the community hospital. Further details can be found in the full study report.[5] Separate topic guides were developed for each group of respondents, each covering broadly similar topics (see supplementary file). The fieldwork took place between November 2015 and February 2017.

Interviews and focus groups were recorded and transcribed verbatim before being imported into NVivo11 software as de-identified files. We conducted thematic analysis, guided by Braun and Clarke's six step process.[19, 20] After an initial reading of transcripts, and a subsequent open coding of a sample of transcripts by three members of the research team, a draft coding frame was developed collaboratively. An initial testing of the coding frame, led to codes being refined, reordered and grouped in to themes, before the final version was applied across the whole dataset. Processes were put in place to ensure consistency of coding across the team, including checking each other's coding practices. The qualitative research team had complementary specialist expertise in both health service management and in voluntary action. Themes were further defined, refined and validated during subsequent stages of analysis and reporting. Emerging findings were discussed with the wider research team and with diverse national and local stakeholders (see PPI). Together these different stages of analysis, throughout which we interacted deeply with the qualitative data, helped to ensure validity and reliability.

After using early findings from the initial quantitative analysis to inform the case study selection, data convergence took place throughout the analysis period, with regular whole-team meetings convened to discuss, triangulate, and integrate emerging findings from across the different stands of analysis. Using quantitative and qualitative methods enabled us to compare and integrate findings from national data on the scale of community support with local, multi-stakeholder perspectives on the forms, role and meaning of support.

For peer review only

Patient and public involvement

Key stakeholders - including members of the Community Hospitals Association, community hospital staff, patients, carers and the public - were involved in all stages of this study, including through a national Steering Group and Local Reference Groups. Emerging findings were discussed at national and local level through Local Reference Group meetings, Annual Learning Events, and conferences. PPI enhanced both the quality, trustworthiness and credibility of the research and its impact.

RESULTS

The study identified four, inter-related, types of support- or 'resource' - that communities provide for their hospitals.

Human resource: giving time through volunteering in community hospitals

Analysis of Charity Commission (CC) records found that Community Hospital Leagues of Friends on average involved 24 volunteers (range 2 to 162): an estimated total of 5,880 volunteers across the 245 community hospitals in the sample. This equates to between 1.4 and 2.5 full time equivalent personnel. If we were to express the value of this using the national minimum wage, the volunteer labour provided by Leagues of Friends equates to a financial input of £15,600–£28,500 per community hospital, or £3.8million to £6.9 million across England. Most case studies involved additional volunteers who were recruited directly by the hospitals, outside of the League of Friends, for which there is no nationally available data.

Our case studies found volunteers tended to be retired, female, white and middle class. Most volunteers become involved through word of mouth, limiting the potential pool of new volunteers. Examples of active volunteer recruitment campaigns were limited. Concerns were raised about the challenge of involving new people. While there were generally high levels of latent support for community hospitals, particularly demonstrable at times of threat to services, this could be difficult to convert into active, regular, volunteer engagement:

For peer review only

'I think that a lot of people would go up in arms if it was closed but whether they'd be willing to do anything about it to help I don't know. There seems to be a little core of people that would but I'm not sure that that spreads right out across the community'

CH9, Carer

In one case study the League of Friends had ceased to operate as volunteers retired and they were unable to recruit new members. In another, however, volunteer involvement had been reinvigorated through the recruitment of a new League of Friends chair and active support from the hospital matron.

Volunteers were involved in a wide range of roles, from fundraising activities, through to delivering activity sessions for patients, supporting meal times, working on a reception desk, and providing a 'hospital to home' befriending service. There was a clear consensus that volunteers could not get involved in medical or personal care or access confidential data and that they were there to complement, not supplement, paid staff. Beyond this, the roles that volunteers undertook varied considerably.

Health and safety regulations and concerns about confidentiality were the most cited restrictions on volunteer involvement. In some, they were seen as reasons not to involve volunteers; in others as appropriate training requirements. Volunteer involvement was further limited by perceptions of key actors within the hospitals concerning: the capacity of patients to engage with volunteers; the views of carers/families towards volunteers; capacity of staff to facilitate volunteering; and the willingness of people to volunteer. Some respondents argued that such concerns were putting *'barriers up where they don't exist'* and that hospitals were not always making the most of *'untapped resource'* available to them.

Financial resources: giving money through fundraising for community hospitals

Leagues of Friends provide substantial financial resource. In 2014, on average, community hospital League of Friends generated financial resources worth £45,387 (Figure 1). This figure is influenced by a small number of very large outliers: the median income was £15,632. In addition, some community hospitals benefit from income generated through

For peer review only

donations made directly to NHS Trusts: these funds are not counted in the above figures (our quantitative data is confined to League of Friends) but were reported to be significant in three case studies.

The community hospital Leagues of Friends were both relatively large and relatively stable entities within their local charitable landscapes: 40% were among the largest 100 charities in their local authorities in terms of income.

Charities generate financial resources for community hospitals through various means. Figure 2 presents an overview of income sources, derived from charity accounts from Leagues of Friends with an income or expenditure greater than £25,000 in any given year. Over a five-year period from 2008-2013, approximately three-quarters (77%) of Leagues of Friends' income was 'voluntary income', that is, income generated through legacies (particularly significant), gifts, donations, grants, membership subscriptions and sponsorships. One-sixth (15%) of the total income was from activities for generating funds, such as jumble sales, lotteries, and charity shops. Less than one-tenth was raised through 'charitable activities', such as trading goods and services.

Figure 3 shows how average levels of the total income and expenditure have varied over time. League of Friends' incomes have been declining since 1995, on average by approximately £901 a year. The decline was most pronounced in 2011 and 2012, showing some signs of recovery in 2013 and 2014. Fixed-effects regression analysis, entering a constant term and year as the only predictors, in order to compare income with that of a base category (income in 1995), indicates that these temporal changes were statistically significant. The average and median income that Leagues of Friends have received yearly was on the rise until the mid-2000s but since then has declined; the decline in income in general has been statistically significant ($b= -901.29$, SE 230.20), $p < .01$; constant $b=1785435$, SE 461467, $p<.001$.

There was, however, little concern amongst our case studies regarding declining levels of income. Indeed, some had limited their fundraising activities, either through recognising a potential crowding out of other local charities or due to uncertainty over the future of the

For peer review only

hospital. Some League of Friend's members, however, questioned how long they could sustain current income levels, often explaining this with reference to declining membership levels. These were also seen as harbingers of more general reduced community support.

Expenditure had also declined, but less sharply than income (Figure 3). Since 1995 community hospital Leagues of Friends had, in most years, received more than they had spent. The exceptions from this trend are years 1997 and 2011 when an average Friends group spent more than it received. In 2011 their funding position was at its weakest (expressed in terms of the gap between income and expenditure) since 1995 (Figure 3).

These financial resources are used to provide various amenities for community hospitals: patient comforts (found across all case studies); staff development; equipment; buildings; staff time (found in a small number of cases). There was uncertainty about where the boundary between voluntary and statutory support should be drawn. Some cases adopted a clear demarcation avoiding anything they felt to be a statutory responsibility (e.g. buildings, equipment, staffing), others did not. Concerns were expressed about the blurring of boundaries:

> *'We are supposed to help the hospital for extras. This was how it started off. It has got to be very much more mainstream now because of lack of funds' CH9, Volunteer*

Practical resources: Providing services through voluntary organisations

Various national and local voluntary organisations also contributed to community hospitals, providing some direct form of service to patients getting into hospital, during their stay, or on discharge. They also provided support for families and carers of hospital patients, or more general support for the hospital through, for example, running fundraising events. Many of these services were free to the hospitals, some were paid for; some were delivered by paid staff employed by the voluntary organisations and others by volunteers. Some voluntary groups, or members of staff from within them, were co-located within the hospital, providing important mutual benefits.

For peer review only

The interaction of community hospitals with such voluntary organisations varied. The general perception was that more could be done to strengthen the links between institutions and local organisations. Two barriers identified were a lack of knowledge of local voluntary and community groups, and a lack of time for hospital staff to identify and build relationships.

Intellectual resources: giving voice through information, consultation and co-production

Leagues of Friends in particular, but also other community groups and individuals, played a role in supporting the hospital through representing the hospital in the community and/or vice versa. This happened at different levels.

At a basic level, an important role for the League of Friends was to provide a communication channel between the hospital and its community. The Leagues of Friends helped raise the profile of the hospital and provide feedback on services. Many League of Friends members were well networked within their local communities, facilitating this role.

Leagues of Friends had come, by design or default, to represent the 'voice of the community' in various consultation processes concerning the future of the hospital/ services. This wasn't always a role they had anticipated, nor was it one which all had the capacity to fulfil. Considerable frustration was expressed about such consultations, focusing on: tokenism, poor processes, lack of timely communication, uneven engagement, and an apparent inability to influence outcomes. Commissioners recognised the importance of consultation, suggesting communities would react '*more reasonably*' to change if they had been involved in decisions. Some commissioners, however, expressed their own frustrations about the consultation processes:

> *'however much you do it's never enough and some people will always feel that they've in some way been excluded.'* (CH8&9, Commissioner)

For peer review only

Deeper, sustained community involvement in the ongoing coproduction of community hospitals services was limited. This led to frustration that opportunities were being missed to draw upon the expertise and energy of the community:

> *'There is no local input at all and I think there should be. You call it a local reference group: I would love to have a small local reference group and that would at least – not run the hospital – but at least be able to have a say and it could be listened to' (CH2, Volunteer)*

Amongst the nine case studies, however, there were two significant exceptions. In one, following a decision by the NHS to close the hospital, the local community established a charity and ran a successful fundraising campaign to re-open it. The charity now owns and runs the hospital, with services provided by the NHS, and has established a range of other health and social care services on the same site. In another, again following (perceived) threats to the future of the hospital, the community formed a local action group which has become a key player in a now well established local health forum which brings together statutory health and social care providers with the local community to co-design and co-deliver services.

Making a difference

Distilling the combined impact of these resources is difficult. Some suggested that community involvement was '*key to the community hospital*': it was what makes a hospital a '*community* hospital'; community hospitals would be a '*totally different place'* without it. In one, however, it was suggested that if the community didn't get involved no-one would really notice, although *'some of the trimmings might disappear round the edge'.* The study identified five sets of outcomes:

- Enhancing community hospital utilisation and resilience: Community engagement, it was suggested, may be an important factor in the apparent resilience of community hospitals.[5] Community involvement had also contributed to service utilisation in some hospitals, through raising awareness of facilities available. However, a

For peer review only

tendency towards resisting change was seen by some to be a downside of community engagement, potentially preventing improvements to service delivery.

- Contributing to patient experience: Enhanced hospital buildings and equipment, resulting from voluntary efforts, were agreed to contribute to patient experience, giving them the *'extra bits which the hospital won't or can't afford',* and contributing to the *'happy atmosphere'* found in most of the case studies. It was suggested that some patients found it easier to talk to volunteers ('*someone out of uniform')* than to staff. Volunteers could help to tackle loneliness and boredom, and more generally to '*lift peoples' spirits'* and '*making them happy'*. Community engagement was also thought to be beneficial at the point of discharge, easing the transition home.

- Boosting staff morale: Staff told us that community involvement had an effect on them. The perception of being supported and valued by the community contributed to an identified distinction between the experience of working in a community hospital and an acute hospital, could help boost staff morale, and build loyalty to the hospital. On the other hand, some staff reported having to invest valuable time in facilitating community engagement, 'pick up' tasks when volunteers had not turned up or had not done a job effectively, and deal with the expectations of community members that arose from their support of the hospital (e.g. an entitlement to access beds).

- Enhancing volunteer well-being: Engaging with the hospital also had outcomes for the volunteers themselves. Volunteering provided structure for some, and acted as a replacement for paid work, or as an alternative to it. The social interaction, physical and mental activity associated with volunteering was important for some, particularly older, respondents. However, for some, volunteering encroached on personal and family time and could be experienced as stressful and tiring, particularly when it proved difficult to recruit enough helpers.

For peer review only

- Community well-being: the activities involved in supporting community hospitals were building, and often reliant upon social interaction, networks and trust. Fundraising events in particular were highlighted as important functions in the social calendar of some communities, bringing people together and tackling social isolation. This was seen as particularly important for older people.

DISCUSSION

Community hospitals are generally well supported by their local communities. Many benefit from considerable input of resources in terms of time, money, service and intelligence. Together through this voluntary 'energy', communities provide significant additional resources to their community hospitals. As previous studies have suggested, these resources can positively affect patient experience[10, 21, 22] and service quality[14] by adding capacity, enhancing facilities, and boosting staff morale.

However, levels of voluntary income have been declining. Clifford likewise found Leagues of Friends were one of the groups of charities that had experienced a decline in income.[23] Membership has also declined. And it was increasingly hard to recruit volunteers. While the study identified widespread latent support for community hospitals, regular, ongoing, active involvement was often limited to a relatively small group of volunteers. Getting new and particularly younger people to actively engage - beyond moments of crisis - was a challenge. Respondents in part attributed these challenges to changes within community hospital services which were felt to make them less accessible or relevant to the local – particularly younger – community[5], and in part to wider societal changes which were generally felt to be making community engagement more difficult (although it is worth noting that long term national levels of volunteering are static[24]). The variance in levels of community support, between and within communities and over time, echoes studies of voluntary support in other areas of healthcare[25, 13] as well as in other sectors.[26, 27] These findings question the extent to which voluntary support can be always be considered a sustainable source of 'renewable energy'.

We agree with Munoz *et al.*'s (p.221) conclusion that *'harnessing more local volunteers […] is more complex than governments assume'.*[25] However, we found limited evidence of

For peer review only

extensive efforts being made to do so. Several limits to the involvement of volunteers and voluntary groups were identified, alongside a general lack of 'investment' in their engagement. Converting the extensive (often passive) support found across communities into more regular, active engagement requires investment of time, enthusiasm and inevitably money. Just as harnessing other sources of renewable energy (e.g. wind, sun) requires investment in the appropriate infrastructure, so too – we suggest - does voluntary and community support. There was evidence from some of our case studies that sustained efforts to encourage and nurture voluntary support could, however, be effective.

This study has provided original insights into voluntary support for community hospitals, demonstrating variations in its contribution, its reliance on a relatively small number of committed individuals, and raises questions as to the extent to which it can be considered sustainable or 'renewable'. This provides important lessons for policies predicated on the expansion of voluntary and community engagement in health care. Communities can be a significant resource for health care services, in ways which can enhance patient experience and service quality, but this resource should not be taken for granted. The scale of voluntary support – volunteering, voluntary income, or co-productive activities – is unlikely to grow without considerable effort at national and local level to support it.

Study limitations include limits to the secondary data available for the detailed analysis of levels of volunteering (Charity Commission records exclude trustees and those who volunteer within community hospitals outside of the League of Friends) and expenditure from charitable funds. The study was also limited by its scope: while we were able to focus in detail on community hospitals, we were not able to fully consider the wider system within which they are embedded.

Future research priorities include longitudinal research to explore changes in the levels and forms of voluntary support and comparative studies of voluntary support in different health care settings.

ETHICAL APROVAL

For peer review only

The Wales Research Ethics Committee 6 reviewed this research study reference: 16/WA/0021, on the 24th February, 2016, and provided a favourable ethical opinion. All research participants gave consent to be involved in the study.

ACKNOWLEDGEMENTS

We would like to thank all our study respondents who contributed to this study through participating in interviews, focus groups, site visits and responding to requests for information. Particular thanks go to matrons/ward sisters, community hospital clinicians and League of Friends members who coordinated research activities for us, organized Local Reference Groups, and participated in learning events. We would also like to acknowledge the inputs of other members of the study team who were involved in the wider study design and data collection but were not directly involved in authoring the article: Nick Le Meseurier, Helen Tucker, John Glasby, Iestyn Williams, David Seamark, Tessa Crilly, John Crilly and Jan Marriot. Thanks also go to our funders, the National Institute for Health Research.

AUTHOR CONTRIBUTIONS

All authors were involved in the writing of the article, with Angela taking the lead. Angela and Deborah led on the qualitative aspects of the study, John and Daiga on the quantitative.

FUNDING

This work was supported by The National Institute for Health Research - Health Services and Delivery Research: New Research on Community Hospitals, grant number 12/177/13.

COMPETING INTERESTS

None

DATA SHARING

The quantitative datasets for the study are available from the UK Data Archive: Alcock, Peter and Mohan, John (2013). *Third Sector Research Centre research data collection.* [Data Collection]. Colchester, Essex: Economic and Social Research Council. 10.5255/UKDA-SN-850933

For peer review only

REFERENCES

1 NHS England. *Five Year Forward View* London: HMSO 2014.

2 NHS England. *The NHS Long Term Plan* London: HMSO 2019.

3 Dudman J. 'The NHS would collapse without them': The growing role of volunteers. *The Guardian,* 2018. https://www.theguardian.com/society/2018/jul/04/nhs-collapse-without-them-growing-role-volunteers (accessed 10.07.19)

4 Crisp, N. What would a sustainable health and care system look like? *BMJ 2017; 358:j3895 doi: 10.1136/bmj.j3895*

5 Davidson D, Ellis Paine A, Glasby J, *et al.* Analysis of the profile, characteristics, patient experience and community value of community hospitals: A multimethod study. *Health Service Delivery Research 2019;*7(1).

6 Hall J. From cottage to community hospitals: Watlington Cottage Hospital and its regional context, 1874-2009. *Local Popul Stud* 2012;88(1):33-49.

7 Sutton C. Axminster Hospital 1886-1986. 1986.

8 Emrys Roberts M. Cottage Hospitals 1859-1990: Arrival, survival and revival. Dorset: Turn Publications 1991.

9 Cooney E. The Leagues of Hospital Friends. *Public Administration* 1960;38:263-272.

10 Naylor C, Mundle C, Weaks L, Buck D. Volunteering in health and care: Securing a sustainable future. London: King's Fund 2013.

11 Mocroft I, Pharoah C. Coming full circle: charitable funds in London's health. London: King's Fund 2001.

12 Lattimer M. The gift of health. London: Directory of Social Change 1996.

13 Galea A, Naylor C, Buck D, Weaks L. Volunteering in acute trusts in England: Understanding the scale and impact. London: The Kings Fund 2013.

14 Mundle C, Naylor C, Buck D. Volunteering in health and care in England. London: The Kings Fund 2012.

15 Creswell J, Creswell, D. *Research design: Qualitative, quantitative and mixed methods approaches.* London: Sage 2018.

16 O'Cathain A, Murphy E, Nicholl J. The quality of mixed methods studies in health services research. *J Health Serv Res Policy* 2008;13(2)92-98. doi: 10.1258/jhsrp.2007.007074.

For peer review only

17 Halaby C. Panel models in sociological research. *Annual Review of Sociology* 2004;30: 507-544. doi.org/10.1146/annurev.soc.30.012703.110629

18 O'Brien B, Harris I, Beckman T et al. Standards for reporting qualitative research: A synthesis of recommendations. *Acad Med*. 2014;89(9):1245-1251. doi: 10.1097/ACM.0000000000000388.

19 Braun V, Clarke V. Using thematic analysis in psychology. *Qual Res Psychol* 2006;3:77-101. doi:10.1191/1478088706qp063oa.

20 Maguire M. and Delahunt B. Doing a thematic analysis: A practical, step-by-step guide for learning and teaching scholars. *AISHE-J* 2017;8(3): 3351-33514.

21 Hotchkiss R, Unruh L, Fottler M. The role, measurement and impact of volunteeerism in hospitals. *Nonprofit and Voluntary Sector Management* 2014;43(6):1111-1128. doi/10.1177/0899764014549057

22 Kang R, Hasnain-Wynia R, Hood M. Hospital commitment to community orientation with its association with quality of care and patient experience. *J Healthc Manag* 2013;58(4): 277-288.

23 Clifford D. Charitable organisations, the great recession and the age of austerity: Longitudinal evidence for England and Wales. *Journal of Social Policy* 2017;46(1): 1-30. doi: 10.1017/S0047279416000325

24 Staetsky L, Mohan, J. *Individual voluntary participation in the United Kingdom: An overview of survey information,* TSRC Working Paper No. 6, Southampton: Third Sector Research Centre 2011.

25 Munoz S-A, Farmer J, Warburton J, Hall J. Involving older people in service co-production: is there an untapped pool of potential participants? *J Rural Stud* 2014;34(3):212-222. doi: 10.1016/j.jrurstud.2014.02.001

26 Mohan J, Bulloch C. *Is there a British 'civic core'? Evidence from the Citizenship Survey.* TSRC Working Paper No. 73; Birmingham: Third Sector Research Centre 2012.

27 Wilson J, Musick M. The Contribution of Social Resources to Volunteering. *Soc Sci Q* 1998;79(4):799–814.

LIST OF FIGURES

Figure 1: League of Friends income distribution in 2014

Figure 2: Community Hospital Leagues of Friends' income sources, by year

For peer review only

Figure 3: Leagues of Friends income and expenditure over time

For peer review only

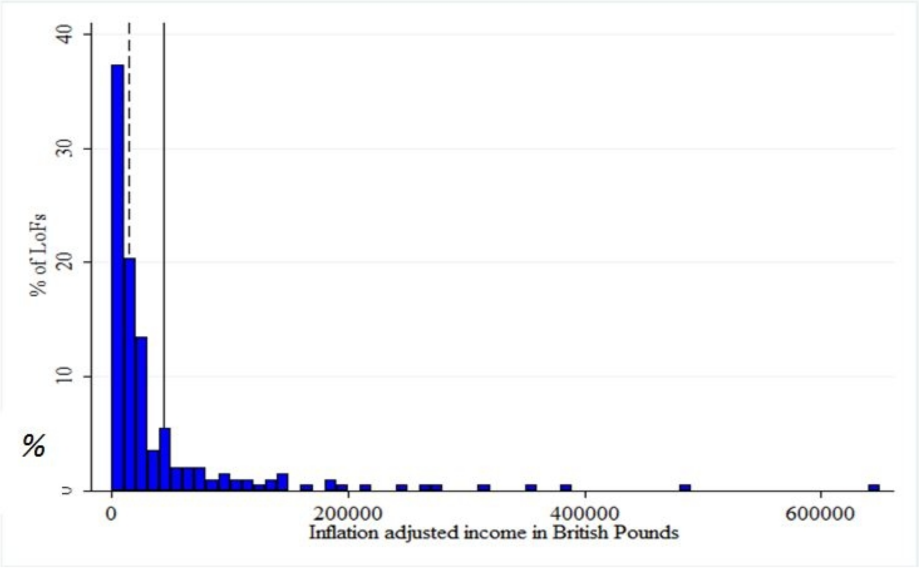

Figure 1: League of Friends income distribution in 2014

Notes: solid line= mean (£45,387); dashed line= median £15,632)

Figure 1: League of Friends income distribution in 2014

168x121mm (120 x 120 DPI)

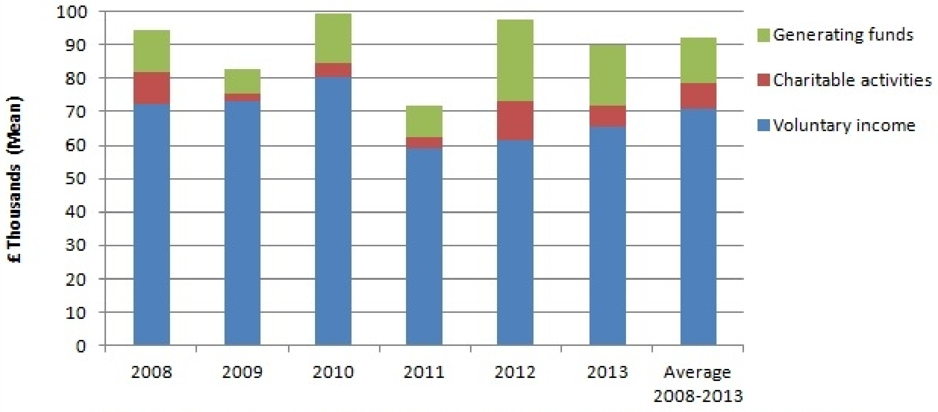

Figure 2: Community Hospital Leagues of Friends' income sources, by year

Figure 2: Community Hospital League of Friends' income sources by year

139x75mm (120 x 120 DPI)

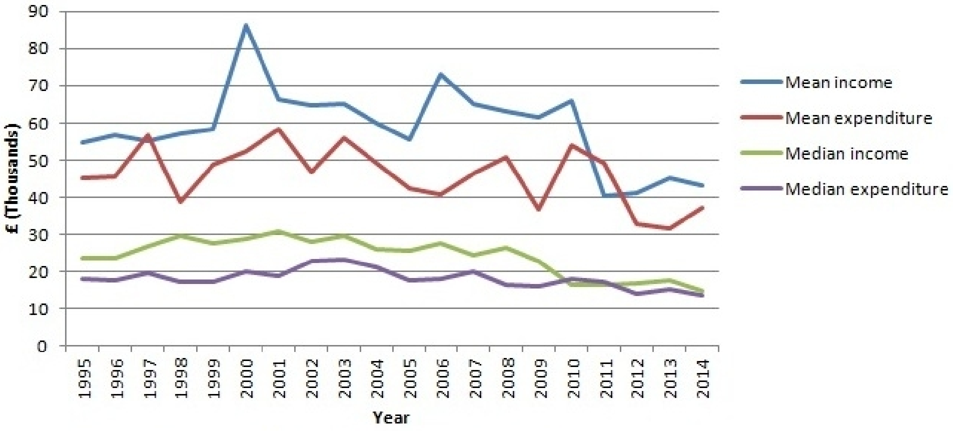

Figure 3: League of Friends income and expenditure over time

Figure 3: League of Friends income and expenditure over time

237x126mm (72 x 72 DPI)

Communities as 'renewable energy' for health care services? A multi-methods study into the form, scale, and role of voluntary support for community hospitals in England

Angela Ellis Paine, Daiga Kamerāde, John Mohan and Deborah Davidson

Supplementary file: Interview topic guide summaries
Community Hospital research study: Profile, patient and carer experience, and community involvement and value

Staff

1) About you
   - Could you briefly tell me about your role in this community hospital?
   - How and why did you come to work at this hospital?
   - What is it like as a place to work?
2) Organisational background
   - Can you give me a basic introduction to this Community Hospital? (prompt for *history, services, staff, volunteers, funding, challenges and opportunities)*
3) Community context
   - Can you tell me a little bit about the local community: what is it like to live/work here?
4) Patient experience
   - How would you describe the patient experience at this community hospital?
   - Tell me about the main groups of patients who use this hospital?
   - What factors do you think are most significant in shaping patient experience?
   - What do you think of the facilities at this hospital? What kind of feedback do you get from the patients/carers about the facilities here?
   - What are the relationships like between patients and staff here?
   - What do you think about the level of information the hospitals provide the patients regarding their care, hospital facilities and wider services?
   - How involved do you think patients are in decisions about their care? In the hospital?
5) Community involvement
   - What are the different ways in which voluntary groups and the local community are involved in the Hospital, as providers, patients and residents? (probe around volunteering, governance, expert patients, services etc)
   - What does the community contribute to the hospital?
   - How is this organised and managed? (probe around recruitment, support, retention, use of external organisations)
   - How would the hospital be affected if the community did not get involved?
   - Do you think there is any relationship between community involvement and patient experience?
6) Value of community hospital to community
   - What does the community hospital contribute to the local community?
   - How would the community be affected if the community hospital wasn't here?
   - What does this community hospital mean to you?

For peer review only

Community stakeholders:

1) About you
    - Could you briefly tell me about you (and your role as [X])?
    - In what ways are you/your organisation involved in the community hospital?
2) Community hospital
    - How would you describe this community hospital?
3) Community context
    - Can you give me a basic introduction to the local community? What is it like to live/work here?
4) Patient experience
    - How would you describe the patient experience at this community hospital?
    - What factors do you think are most significant in shaping patient experience? (e.g. facilities, relationships, information, involvement)
5) Community involvement
    - What are the different ways in which voluntary groups and the local community are involved in the Hospital, as providers, patients and residents? (probe around volunteering, governance, expert patients, services etc)
    - What does the community contribute to the hospital?
    - How is this organised and managed? (probe around recruitment, support, retention, use of external organisations)
    - How would the hospital be affected if the community did not get involved?
    - Is there a connection between community involvement and patient experience?
6) Value of community hospital to community
    - What does the community hospital contribute to the local community?
    - How would the community be affected if the community hospital wasn't here?
    - What does the community hospital mean to you?

Volunteers

1) About you
    - Can you tell us a little bit about yourself, and about your role as a volunteer at [this] community hospital?
2) Getting involved
    - How did you come to be involved as a volunteer with the hospital? (prompt around being involved through another organisation, receiving services, or connections through family etc)
    - What motivated you to get involved?
3) Volunteering role
    - Can you tell us a bit more about what your role as a volunteer here? What does a typical day as a CH volunteer involve?
    - Has your role evolved over the course of the involvement in the CH?
    - Have you been able to do the kind of volunteer-roles that you hoped? If not, why not?
    - How frequently do you volunteer for the CH?
4) Volunteer support
    - What kind of support have you received as a volunteer at the hospital? (prompt for induction, training, supervision)

For peer review only

- How would you describe the relationship between volunteers and paid staff at the hospital?
- Overall, how helpful do you feel the support for volunteers is at the hospital? What is particularly good? What could be improved?

5) Volunteering outcomes
   - What difference do volunteers make to the community hospital as a whole?
   - What impact do volunteers have on the staff who work here?
   - What difference does the role of volunteers make to hospital patients, and carers? Can you give me any specific examples?
   - Is there anything that could be done differently to strengthen the outcomes for service users?
   - What difference has being involved as a volunteer at the CH made to you? (prompt for skills; friendships; enjoyment; employability)
   - Is there anything you don't like about being a volunteer here?
6) Value of community hospital to community
   - What does the community hospital contribute to the local community?
   - How would the community be affected if the community hospital wasn't here?
   - What does this community hospital mean to you?

Carer:

1) About you
   - Could you briefly tell me about you and your relationship to [patient]?
2) Services
   - What are the main services that you have had experience of through being a carer for a patient at this hospital?
3) Carer experience
   - Can you tell us about your experience of being a carer of a patient at [X] community hospital? (prompt for: care, support, treatment, facilities, relationships, involvement, feelings)
   - Was there anything that was particularly good about being a carer of a patient here?
   - Was there anything that was particularly difficult about being a carer of a patient here?
   - Was there anything that could have been done better to improve your experience of being a carer of a patient at [x] Community Hospital?
4) Community involvement
   - Have you been involved in the CH in any other way, such as being a patient, working here or being a volunteer?
   - How involved do you think the local community is in the hospital?
   - How would the hospital be affected if the community did not get involved?
5) Value of community hospital to community
   - What does the community hospital contribute to the local community?
   - How would the community be affected if the community hospital wasn't here?
   - What does the community hospital mean to you?

For peer review only

Patients (discovery interview)

Part A

1) We would like to hear about your experiences of this [name] community hospital. You may find it helpful to look at this picture and think about different elements of your experiences and factors that may have shaped them.

Prompts, if not covered in story, for: facilities, relationships, involvement in decisions, feelings

Part B

1) Was there anything that was particularly good about your experience of being a patient at [X] Community Hospital?
2) Was there anything that wasn't particularly good about your experience of being a patient at [x] Community Hospital?
3) What would have improved your experience of being a patient at [x] Community Hospital?
4) Have you ever been involved in the hospital in any way other than as a patient, such as being a member of staff, or a volunteer?
5) Finally a question we are asking everyone we speak with is "What does the Community Hospital mean to you?"

For peer review only

Reporting checklist for qualitative study.

Based on the SRQR guidelines.

Instructions to authors

Complete this checklist by entering the page numbers from your manuscript where readers will find each of the items listed below.

Your article may not currently address all the items on the checklist. Please modify your text to include the missing information. If you are certain that an item does not apply, please write "n/a" and provide a short explanation.

Upload your completed checklist as an extra file when you submit to a journal.

In your methods section, say that you used the SRQRreporting guidelines, and cite them as:

O'Brien BC, Harris IB, Beckman TJ, Reed DA, Cook DA. Standards for reporting qualitative research: a synthesis of recommendations. Acad Med. 2014;89(9):1245-1251.

		Reporting Item	Page Number
**Title**			
	#1	Concise description of the nature and topic of the study identifying the study as qualitative or indicating the approach (e.g. ethnography, grounded theory) or data collection methods (e.g. interview, focus group) is recommended	1&2
**Abstract**			

For peer review only

	#2	Summary of the key elements of the study using the abstract format of the intended publication; typically includes background, purpose, methods, results and conclusions	1&2
**Introduction**			
Problem formulation	#3	Description and signifcance of the problem / phenomenon studied: review of relevant theory and empirical work; problem statement	3-4
Purpose or research question	#4	Purpose of the study and specific objectives or questions	3-5
**Methods**			
Qualitative approach and research paradigm	#5	Qualitative approach (e.g. ethnography, grounded theory, case study, phenomenolgy, narrative research) and guiding theory if appropriate; identifying the research paradigm (e.g. postpositivist, constructivist / interpretivist) is also recommended; rationale. The rationale should briefly discuss the justification for choosing that theory, approach, method or technique rather than other options available; the assumptions and limitations implicit in those choices and how those choices influence study conclusions and transferability. As appropriate	6-7

For peer review only

		the rationale for several items might be discussed together.	
Researcher characteristics and reflexivity	#6	Researchers' characteristics that may influence the research, including personal attributes, qualifications / experience, relationship with participants, assumptions and / or presuppositions; potential or actual interaction between researchers' characteristics and the research questions, approach, methods, results and / or transferability	7
Context	#7	Setting / site and salient contextual factors; rationale	6-7
Sampling strategy	#8	How and why research participants, documents, or events were selected; criteria for deciding when no further sampling was necessary (e.g. sampling saturation); rationale	7
Ethical issues pertaining to human subjects	#9	Documentation of approval by an appropriate ethics review board and participant consent, or explanation for lack thereof; other confidentiality and data security issues	17
Data collection methods	#10	Types of data collected; details of data collection procedures including (as appropriate) start and stop dates of data collection and analysis, iterative process, triangulation of	5-8

For peer review only

		sources / methods, and modification of procedures in response to evolving study findings; rationale	
Data collection instruments and technologies	#11	Description of instruments (e.g. interview guides, questionnaires) and devices (e.g. audio recorders) used for data collection; if / how the instruments(s) changed over the course of the study	7 (with topics guides as supplementary doc)
Units of study	#12	Number and relevant characteristics of participants, documents, or events included in the study; level of participation (could be reported in results)	7
Data processing	#13	Methods for processing data prior to and during analysis, including transcription, data entry, data management and security, verification of data integrity, data coding, and anonymisation / deidentification of excerpts	7
Data analysis	#14	Process by which inferences, themes, etc. were identified and developed, including the researchers involved in data analysis; usually references a specific paradigm or approach; rationale	7

For peer review only

Techniques to enhance trustworthiness	#15	Techniques to enhance trustworthiness and credibility of data analysis (e.g. member checking, audit trail, triangulation); rationale	7-8

Results/findings

Syntheses and interpretation	#16	Main findings (e.g. interpretations, inferences, and themes); might include development of a theory or model, or integration with prior research or theory	8-15
Links to empirical data	#17	Evidence (e.g. quotes, field notes, text excerpts, photographs) to substantiate analytic findings	8-15

Discussion

Intergration with prior work, implications, transferability and contribution(s) to the field	#18	Short summary of main findings; explanation of how findings and conclusions connect to, support, elaborate on, or challenge conclusions of earlier scholarship; discussion of scope of application / generalizability; identification of unique contributions(s) to scholarship in a discipline or field	15-16
Limitations	#19	Trustworthiness and limitations of findings	8, 16

Other

For peer review only

Conflicts of interest	#20	Potential sources of influence of perceived influence on study conduct and conclusions; how these were managed	17
Funding	#21	Sources of funding and other support; role of funders in data collection, interpretation and reporting	17

None The SRQR checklist is distributed with permission of Wolters Kluwer © 2014 by the Association of American Medical Colleges. This checklist can be completed online using https://www.goodreports.org/, a tool made by the EQUATOR Network in collaboration with Penelope.ai

For peer review only

Communities as ‘renewable energy’ for health care services? A multi-methods study into the form, scale and role of voluntary support for community hospitals in England

Journal:	*BMJ Open*
Manuscript ID	bmjopen-2019-030243.R2
Article Type:	Original research
Date Submitted by the Author:	14-Aug-2019
Complete List of Authors:	Ellis Paine, Angela; University of Birmingham, Third Sector Research Centre, School of Social Policy<br>Kamerāde, Daiga; University of Salford, School of Health and Society<br>Mohan, John; University of Birmingham, Third Sector Research Center (TSRC)<br>Davidson, Deborah; University of Birmingham, Health Services Management Centre
**Primary Subject Heading**:	Health services research
Secondary Subject Heading:	Qualitative research, Health policy
Keywords:	community hospital, volunteering, voluntary income, co-production, change over time, sustainability

Communities as 'renewable energy' for health care services? A multi-methods study into the form, scale, and role of voluntary support for community hospitals in England

Angela Ellis Paine, Daiga Kamerāde, John Mohan and Deborah Davidson

Corresponding author:

Angela Ellis Paine, University of Birmingham

Postal address: Third Sector Research Centre, Room 1151 Muirhead Tower, University of Birmingham, Edgbaston, Birmingham, B15 2TT

Email: a.ellispaine@bham.ac.uk

Phone: 07753747883

ORCID ID: 0000-0002-4385-5098

Co-authors:

Daiga Kamerāde, School of Health and Society, Salford University, Salford, UK. Email: D.Kamerade-Hanta@salford.ac.uk

John Mohan, Third Sector Research Centre, School of Social Policy, University of Birmingham, Birmingham, UK. Email: j.mohan@bham.ac.uk

Deborah Davidson, Health Services Management Centre, School of Social Policy, University of Birmingham, Birmingham, UK. Email: d.c.davidson@bham.ac.uk

Article word count: 4,274

For peer review only

Communities as 'renewable energy' for health care services? A multi-methods study into the form, scale and role of voluntary support for community hospitals in England

KEY WORDS

Community hospitals, volunteering, voluntary income, co-production, sustainability

ABSTRACT

Objective

To examine the forms, scale and role of community and voluntary support for community hospitals in England.

Design

A multi-methods study. Quantitative analysis of Charity Commission data on levels of volunteering and voluntary income for charities supporting community hospitals. Nine qualitative case studies of community hospitals and their surrounding communities, including interviews and focus groups.

Setting

Community hospitals in England and their surrounding communities.

Participants

Charity Commission data for 245 community hospital Leagues of Friends. Interviews with staff (89), patients (60), carers (28), volunteers (35), community representatives (20), managers and commissioners (9). Focus groups with multi-disciplinary teams (8 groups across nine sites, involving 43 respondents), volunteers (6 groups, 33 respondents) and community stakeholders (8 groups, 54 respondents).

Results

Communities support community hospitals through: *human resources* (average = 24 volunteers a year per hospital); *financial resources* (median voluntary income = £15,632); *practical resources* through services and activities provided by voluntary and community groups; and *intellectual resources* (e.g. consultation and coproduction). Communities provide valuable supplementary resources to the NHS, enhancing community hospital services, patient experience, staff morale and volunteer well-being. Such resources, however, vary in level and form from hospital to hospital and over time: voluntary income is

For peer review only

on the decline, as is membership of League of Friends, and it can be hard to recruit regular, active volunteers.

Conclusions

Communities can be a significant resource for health care services, in ways which can enhance patient experience and service quality. Harnessing that resource, however, is not straight forward and there is a perception that it might be becoming more difficult questioning the extent to which it can be considered sustainable or ‘renewable’.

ARTICLE SUMMARY: STRENGTHS AND LIMITATIONS

- A robust multi-methods study allowing us to understand the breadth, depth and variability of community support for community hospitals.
- Original insights and data on the levels and forms of voluntary support for community hospitals, which had previously been almost non-existent.
- Limited by the availability of secondary data for the detailed analysis of levels of volunteering (particularly outside of Leagues of Friends) and expenditure from charitable funds.
- Limited by its scope to consider the wider system within which community hospitals are embedded.

For peer review only

INTRODUCTION

A growing recognition of a need to find new ways to deliver public services has contributed to an increasing emphasis on involving communities and individual volunteers in the NHS. NHS England's Five Year Forward View, which in 2014 set out a vision for the future of the NHS, identified communities and patients as 'renewable energy'.[1] This suggests they are seen as a sustainable resource that can be naturally replenished to support the NHS. More recently, NHS England's 'NHS Long Term Plan' has committed to supporting the aim of doubling the number of volunteers in the NHS.[2] Some have gone as far as to suggest the NHS would collapse without the support of volunteers.[3] More broadly, Crisp identifies voluntary and community organisations as part of the 'informal system of care', which, if strengthened, could relieve pressure on health and social care services, contributing to their sustainability.[4] Such claims, however, are often made with limited evidence. We know little about how extensive community involvement is within the NHS, what form it takes, how it varies, or to what extent this 'energy' can be considered sustainable or 'renewable'.

Community hospitals are a long standing part of the healthcare landscape. In 2015, there were 296 community hospitals in England. Community hospitals are small (typically less than 30 beds), predominantly rural, traditionally GP-led, and provide a varied mix of intermediate care services.[5] They often originated as cottage hospitals through local voluntary initiative.[6] Under NHS control, the traditions of voluntary support were maintained, particularly through the formation of Leagues of Friends – independent charities set up to raise funds and mobilise support. Historical accounts have highlighted substantial local commitment to individual community hospitals[6-8], but we are aware of only one academic study of Hospital Leagues of Friends in the UK, published in 1960.[9]

Existing national survey evidence provides only a very general insight into voluntary action in the NHS. Naylor *et al.* estimate that approximately 2.9 million people in England volunteer for health-related organisations and causes, but cannot break this down by specific settings.[10] Studies of voluntary income in healthcare have focused on relatively large organisations[11] or looked at data across authorities.[12] Galea *et al*'s study of volunteering in NHS Acute (secondary care) Trusts in England found that on average they

involve 471 volunteers each.[13] The study highlighted variations between Trusts, but focused only on one form of voluntary support in one particular setting.

A small number of studies have considered the outcomes of certain forms of community engagement for hospitals and for the wider healthcare system. There is some evidence that volunteers can have a positive impact on healthcare, through improving patient experience, strengthening the relationships between services and community, improving public health and supporting integrated care.[10,14] Such evidence, however, is limited.

To investigate how communities support their community hospitals we draw on a multi-methods study. The study offers original evidence on the scale, form and role of voluntary support for community hospitals, and variations over time. Given current and likely future pressures on resources within the NHS, we reflect upon the extent to which voluntary support may be considered a sustainable form of 'renewable energy' offering important additional resources for hospitals, patients and communities.

METHODS

We draw on evidence from a large multi-methods study exploring the profile, characteristics, patient experience, community engagement and value of community hospitals.[5] One aim of this wider study – and of this paper – was to address the question: 'what do communities do to support their community hospitals?' More specifically, the intention is to explore the scale, forms, role and character of voluntary support to community hospitals and how these have changed over time. Developing a complete understanding of voluntary support required a mix of both quantitative (how much) and qualitative (how and why) data. A convergent design was employed, with the qualitative and quantitative elements conducted broadly in parallel and given equal status. Different researchers were involved in the two elements. The research followed good practice guidelines on mixed methods studies.[15, 16]

The quantitative element involved examining the scale of volunteering and financial support for community hospitals through compiling and analysing a dataset of records held by the Charity Commission (the charity regulator in England and Wales). The sample for this study

For peer review only

was 245 Leagues of Friends (and other similar charities) in England that directly support community hospitals and for which financial information was available for at least one year between 1995 and 2014. The Charity Commission records included data on the finances and volunteer numbers for each charity.

We conducted a more detailed analysis of income sources and types of expenditure using data captured from the annual financial accounts of those charities whose income or expenditure exceeds the threshold (£25,000 per year) above which financial accounts are made available on the Charity Commission website. This gave data covering 358 sets of accounts across six financial years; the average number of charities with detailed accounts was 60 per financial year. At the time of conducting analysis, the latest detailed financial information available was for the financial year 2013. The number of accounts available varies from year to year because an individual charity may or may not exceed the £25,000 threshold, depending on fluctuations in its finances. All financial figures were adjusted for inflation using the Office for National Statistics Retail Prices annual index; therefore all financial information in this paper is presented in 2014 prices.

We undertook descriptive analysis of variations and used a fixed effects regression model[17] to examine how within-hospital charity changes in time (years) are linked to changes in the annual income within each individual charity over time, while eliminating unobserved heterogeneity – confounding effects from time-constant variables. The year variable examines any variation in the outcome that happen over time.

The qualitative element involved adopting a case study approach to explore the form, role, experiences and perceived outcomes of community support, reported here using the SRQR guidelines.[18] Nine case studies were selected from a database of community hospitals in England, reflecting diversity in terms of: location, size, models of ownership and provision, and levels of voluntary income (from well below average to well above).

Each case study involved: scoping; a local reference group (staff and community members brought together to inform the study and reflect on emerging findings); semi-structured interviews with: Trust managers and commissioners (9, across the 9 cases), staff (89),

For peer review only - http://bmjopen.bmj.com/site/about/guidelines.xhtml

volunteers (35), community stakeholders (20) and carers (28); discovery interviews with patients (60); focus groups with multi-disciplinary teams (8, across the 9 sites, involving 43 respondents), volunteers (6 groups, 33 respondents) and community stakeholders (8 groups, 54 respondents). We used purposive sampling, informed by our scoping visits, local reference groups and snowball techniques, with respondents selected due to their involvement in or knowledge of the community hospital. Further details can be found in the full study report.[5] Separate topic guides were developed for each group of respondents, each covering broadly similar topics (see supplementary file). The fieldwork took place between November 2015 and February 2017.

Interviews and focus groups were recorded and transcribed verbatim before being imported into NVivo11 software as de-identified files. We conducted thematic analysis, guided by Braun and Clarke's six step process.[19, 20] After an initial reading of transcripts, and a subsequent open coding of a sample of transcripts by three members of the research team, a draft coding frame was developed collaboratively. An initial testing of the coding frame, led to codes being refined, reordered and grouped in to themes, before the final version was applied across the whole dataset. Processes were put in place to ensure consistency of coding across the team, including checking each other's coding practices. The qualitative research team had complementary specialist expertise in both health service management and in voluntary action. Themes were further defined, refined and validated during subsequent stages of analysis and reporting. Emerging findings were discussed with the wider research team and with diverse national and local stakeholders (see PPI). Together these different stages of analysis, throughout which we interacted deeply with the qualitative data, helped to ensure validity and reliability.

After using early findings from the initial quantitative analysis to inform the case study selection, data convergence took place throughout the analysis period, with regular whole-team meetings convened to discuss, triangulate, and integrate emerging findings from across the different stands of analysis. Using quantitative and qualitative methods enabled us to compare and integrate findings from national data on the scale of community support with local, multi-stakeholder perspectives on the forms, role and meaning of support.

For peer review only

Patient and public involvement

Key stakeholders - including members of the Community Hospitals Association, community hospital staff, patients, carers and the public - were involved in all stages of this study, including through a national Steering Group and Local Reference Groups. Emerging findings were discussed at national and local level through Local Reference Group meetings, Annual Learning Events, and conferences. PPI enhanced both the quality, trustworthiness and credibility of the research and its impact.

RESULTS

The study identified four, inter-related, types of support- or 'resource' - that communities provide for their hospitals.

Human resource: giving time through volunteering in community hospitals

Analysis of Charity Commission (CC) records found that Community Hospital Leagues of Friends on average involved 24 volunteers (range 2 to 162): an estimated total of 5,880 volunteers across the 245 community hospitals in the sample. This equates to between 1.4 and 2.5 full time equivalent personnel. If we were to express the value of this using the national minimum wage, the volunteer labour provided by Leagues of Friends equates to a financial input of £15,600–£28,500 per community hospital, or £3.8million to £6.9 million across England. Most case studies involved additional volunteers who were recruited directly by the hospitals, outside of the League of Friends, for which there is no nationally available data.

Our case studies found volunteers tended to be retired, female, white and middle class. Most volunteers become involved through word of mouth, limiting the potential pool of new volunteers. Examples of active volunteer recruitment campaigns were limited. Concerns were raised about the challenge of involving new people. While there were generally high levels of latent support for community hospitals, particularly demonstrable at times of threat to services, this could be difficult to convert into active, regular, volunteer engagement:

For peer review only

'I think that a lot of people would go up in arms if it was closed but whether they'd be willing to do anything about it to help I don't know. There seems to be a little core of people that would but I'm not sure that that spreads right out across the community'

CH9, Carer

In one case study the League of Friends had ceased to operate as volunteers retired and they were unable to recruit new members. In another, however, volunteer involvement had been reinvigorated through the recruitment of a new League of Friends chair and active support from the hospital matron.

Volunteers were involved in a wide range of roles, from fundraising activities, through to delivering activity sessions for patients, supporting meal times, working on a reception desk, and providing a 'hospital to home' befriending service. There was a clear consensus that volunteers could not get involved in medical or personal care or access confidential data and that they were there to complement, not supplement, paid staff. Beyond this, the roles that volunteers undertook varied considerably.

Health and safety regulations and concerns about confidentiality were the most cited restrictions on volunteer involvement. In some, they were seen as reasons not to involve volunteers; in others as appropriate training requirements. Volunteer involvement was further limited by perceptions of key actors within the hospitals concerning: the capacity of patients to engage with volunteers; the views of carers/families towards volunteers; capacity of staff to facilitate volunteering; and the willingness of people to volunteer. Some respondents argued that such concerns were putting *'barriers up where they don't exist'* and that hospitals were not always making the most of *'untapped resource'* available to them.

Financial resources: giving money through fundraising for community hospitals

Leagues of Friends provide substantial financial resource. In 2014, on average, community hospital League of Friends generated financial resources worth £45,387 (Figure 1). This figure is influenced by a small number of very large outliers: the median income was £15,632. In addition, some community hospitals benefit from income generated through

For peer review only

donations made directly to NHS Trusts: these funds are not counted in the above figures (our quantitative data is confined to League of Friends) but were reported to be significant in three case studies.

The community hospital Leagues of Friends were both relatively large and relatively stable entities within their local charitable landscapes: 40% were among the largest 100 charities in their local authorities in terms of income.

Charities generate financial resources for community hospitals through various means. Figure 2 presents an overview of income sources, derived from charity accounts from Leagues of Friends with an income or expenditure greater than £25,000 in any given year. Over a five-year period from 2008-2013, approximately three-quarters (77%) of Leagues of Friends' income was 'voluntary income', that is, income generated through legacies (particularly significant), gifts, donations, grants, membership subscriptions and sponsorships. One-sixth (15%) of the total income was from activities for generating funds, such as jumble sales, lotteries, and charity shops. Less than one-tenth was raised through 'charitable activities', such as trading goods and services.

Figure 3 shows how average levels of the total income and expenditure have varied over time. League of Friends' incomes have been declining since 1995, on average by approximately £901 a year. The decline was most pronounced in 2011 and 2012, showing some signs of recovery in 2013 and 2014. Fixed-effects regression analysis, entering a constant term and year as the only predictors, in order to compare income with that of a base category (income in 1995), indicates that these temporal changes were statistically significant. The average and median income that Leagues of Friends have received yearly was on the rise until the mid-2000s but since then has declined; the decline in income in general has been statistically significant ($b= -901.29$, SE 230.20), $p < .01$; constant $b=1785435$, SE 461467, $p<.001$.

There was, however, little concern amongst our case studies regarding declining levels of income. Indeed, some had limited their fundraising activities, either through recognising a potential crowding out of other local charities or due to uncertainty over the future of the

For peer review only

hospital. Some League of Friend's members, however, questioned how long they could sustain current income levels, often explaining this with reference to declining membership levels. These were also seen as harbingers of more general reduced community support.

Expenditure had also declined, but less sharply than income (Figure 3). Since 1995 community hospital Leagues of Friends had, in most years, received more than they had spent. The exceptions from this trend are years 1997 and 2011 when an average Friends group spent more than it received. In 2011 their funding position was at its weakest (expressed in terms of the gap between income and expenditure) since 1995 (Figure 3).

These financial resources are used to provide various amenities for community hospitals: patient comforts (found across all case studies); staff development; equipment; buildings; staff time (found in a small number of cases). There was uncertainty about where the boundary between voluntary and statutory support should be drawn. Some cases adopted a clear demarcation avoiding anything they felt to be a statutory responsibility (e.g. buildings, equipment, staffing), others did not. Concerns were expressed about the blurring of boundaries:

> *'We are supposed to help the hospital for extras. This was how it started off. It has got to be very much more mainstream now because of lack of funds' CH9, Volunteer*

Practical resources: Providing services through voluntary organisations

Various national and local voluntary organisations also contributed to community hospitals, providing some direct form of service to patients getting into hospital, during their stay, or on discharge. They also provided support for families and carers of hospital patients, or more general support for the hospital through, for example, running fundraising events. Many of these services were free to the hospitals, some were paid for; some were delivered by paid staff employed by the voluntary organisations and others by volunteers. Some voluntary groups, or members of staff from within them, were co-located within the hospital, providing important mutual benefits.

For peer review only

The interaction of community hospitals with such voluntary organisations varied. The general perception was that more could be done to strengthen the links between institutions and local organisations. Two barriers identified were a lack of knowledge of local voluntary and community groups, and a lack of time for hospital staff to identify and build relationships.

Intellectual resources: giving voice through information, consultation and co-production

Leagues of Friends in particular, but also other community groups and individuals, played a role in supporting the hospital through representing the hospital in the community and/or vice versa. This happened at different levels.

At a basic level, an important role for the League of Friends was to provide a communication channel between the hospital and its community. The Leagues of Friends helped raise the profile of the hospital and provide feedback on services. Many League of Friends members were well networked within their local communities, facilitating this role.

Leagues of Friends had come, by design or default, to represent the 'voice of the community' in various consultation processes concerning the future of the hospital/ services. This wasn't always a role they had anticipated, nor was it one which all had the capacity to fulfil. Considerable frustration was expressed about such consultations, focusing on: tokenism, poor processes, lack of timely communication, uneven engagement, and an apparent inability to influence outcomes. Commissioners recognised the importance of consultation, suggesting communities would react '*more reasonably*' to change if they had been involved in decisions. Some commissioners, however, expressed their own frustrations about the consultation processes:

> *'however much you do it's never enough and some people will always feel that they've in some way been excluded.'* (CH8&9, Commissioner)

For peer review only - http://bmjopen.bmj.com/site/about/guidelines.xhtml

Deeper, sustained community involvement in the ongoing coproduction of community hospitals services was limited. This led to frustration that opportunities were being missed to draw upon the expertise and energy of the community:

> *'There is no local input at all and I think there should be. You call it a local reference group: I would love to have a small local reference group and that would at least – not run the hospital – but at least be able to have a say and it could be listened to' (CH2, Volunteer)*

Amongst the nine case studies, however, there were two significant exceptions. In one, following a decision by the NHS to close the hospital, the local community established a charity and ran a successful fundraising campaign to re-open it. The charity now owns and runs the hospital, with services provided by the NHS, and has established a range of other health and social care services on the same site. In another, again following (perceived) threats to the future of the hospital, the community formed a local action group which has become a key player in a now well established local health forum which brings together statutory health and social care providers with the local community to co-design and co-deliver services.

Making a difference

Distilling the combined impact of these resources is difficult. Some suggested that community involvement was '*key to the community hospital*': it was what makes a hospital a '*community* hospital'; community hospitals would be a *'totally different place'* without it. In one, however, it was suggested that if the community didn't get involved no-one would really notice, although *'some of the trimmings might disappear round the edge'.* The study identified five sets of outcomes:

- Enhancing community hospital utilisation and resilience: Community engagement, it was suggested, may be an important factor in the apparent resilience of community hospitals.[5] Community involvement had also contributed to service utilisation in some hospitals, through raising awareness of facilities available. However, a

For peer review only

tendency towards resisting change was seen by some to be a downside of community engagement, potentially preventing improvements to service delivery.

- Contributing to patient experience: Enhanced hospital buildings and equipment, resulting from voluntary efforts, were agreed to contribute to patient experience, giving them the *'extra bits which the hospital won't or can't afford',* and contributing to the *'happy atmosphere'* found in most of the case studies. It was suggested that some patients found it easier to talk to volunteers ('*someone out of uniform')* than to staff. Volunteers could help to tackle loneliness and boredom, and more generally to '*lift peoples' spirits'* and '*making them happy*'. Community engagement was also thought to be beneficial at the point of discharge, easing the transition home.

- Boosting staff morale: Staff told us that community involvement had an effect on them. The perception of being supported and valued by the community contributed to an identified distinction between the experience of working in a community hospital and an acute hospital, could help boost staff morale, and build loyalty to the hospital. On the other hand, some staff reported having to invest valuable time in facilitating community engagement, 'pick up' tasks when volunteers had not turned up or had not done a job effectively, and deal with the expectations of community members that arose from their support of the hospital (e.g. an entitlement to access beds).

- Enhancing volunteer well-being: Engaging with the hospital also had outcomes for the volunteers themselves. Volunteering provided structure for some, and acted as a replacement for paid work, or as an alternative to it. The social interaction, physical and mental activity associated with volunteering was important for some, particularly older, respondents. However, for some, volunteering encroached on personal and family time and could be experienced as stressful and tiring, particularly when it proved difficult to recruit enough helpers.

For peer review only

- Community well-being: the activities involved in supporting community hospitals were building, and often reliant upon social interaction, networks and trust. Fundraising events in particular were highlighted as important functions in the social calendar of some communities, bringing people together and tackling social isolation. This was seen as particularly important for older people.

DISCUSSION

Community hospitals are generally well supported by their local communities. Many benefit from considerable input of resources in terms of time, money, service and intelligence. Together through this voluntary 'energy', communities provide significant additional resources to their community hospitals. As previous studies have suggested, these resources can positively affect patient experience[10, 21, 22] and service quality[14] by adding capacity, enhancing facilities, and boosting staff morale.

However, levels of voluntary income have been declining. Clifford likewise found Leagues of Friends were one of the groups of charities that had experienced a decline in income.[23] Membership has also declined. And it was increasingly hard to recruit volunteers. While the study identified widespread latent support for community hospitals, regular, ongoing, active involvement was often limited to a relatively small group of volunteers. Getting new and particularly younger people to actively engage - beyond moments of crisis - was a challenge. Respondents in part attributed these challenges to changes within community hospital services which were felt to make them less accessible or relevant to the local – particularly younger – community[5], and in part to wider societal changes which were generally felt to be making community engagement more difficult (although it is worth noting that long term national levels of volunteering are static[24]). The variance in levels of community support, between and within communities and over time, echoes studies of voluntary support in other areas of healthcare[25, 13] as well as in other sectors.[26, 27] These findings question the extent to which voluntary support can be always be considered a sustainable source of 'renewable energy'.

We agree with Munoz *et al.*'s (p.221) conclusion that *'harnessing more local volunteers […] is more complex than governments assume'.*[25] However, we found limited evidence of

For peer review only

extensive efforts being made to do so. Several limits to the involvement of volunteers and voluntary groups were identified, alongside a general lack of 'investment' in their engagement. Converting the extensive (often passive) support found across communities into more regular, active engagement requires investment of time, enthusiasm and inevitably money. Just as harnessing other sources of renewable energy (e.g. wind, sun) requires investment in the appropriate infrastructure, so too – we suggest - does voluntary and community support. There was evidence from some of our case studies that sustained efforts to encourage and nurture voluntary support could, however, be effective.

This study has provided original insights into voluntary support for community hospitals, demonstrating variations in its contribution, its reliance on a relatively small number of committed individuals, and raises questions as to the extent to which it can be considered sustainable or 'renewable'. This provides important lessons for policies predicated on the expansion of voluntary and community engagement in health care. Communities can be a significant resource for health care services, in ways which can enhance patient experience and service quality, but this resource should not be taken for granted. The scale of voluntary support – volunteering, voluntary income, or co-productive activities – is unlikely to grow without considerable effort at national and local level to support it.

Study limitations include limits to the secondary data available for the detailed analysis of levels of volunteering (Charity Commission records exclude trustees and those who volunteer within community hospitals outside of the League of Friends) and expenditure from charitable funds. The study was also limited by its scope: while we were able to focus in detail on community hospitals, we were not able to fully consider the wider system within which they are embedded.

Future research priorities include longitudinal research to explore changes in the levels and forms of voluntary support and comparative studies of voluntary support in different health care settings.

ETHICAL APROVAL

For peer review only

The Wales Research Ethics Committee 6 reviewed and approved this research study on the 24th February, 2016, reference: 16/WA/0021. All research participants gave consent to be involved in the study.

ACKNOWLEDGEMENTS

We would like to thank all our study respondents who contributed to this study through participating in interviews, focus groups, site visits and responding to requests for information. Particular thanks go to matrons/ward sisters, community hospital clinicians and League of Friends members who coordinated research activities for us, organized Local Reference Groups, and participated in learning events. We would also like to acknowledge the inputs of other members of the study team who were involved in the wider study design and data collection but were not directly involved in authoring the article: Nick Le Meseurier, Helen Tucker, John Glasby, Iestyn Williams, David Seamark, Tessa Crilly, John Crilly and Jan Marriot. Thanks also go to our funders, the National Institute for Health Research.

AUTHOR CONTRIBUTIONS

All authors meet the ICMJE criteria for authorship. All made substantial contributions to the design and analysis: Angela and Deborah led on the qualitative aspects of the study, Daiga and John on the quantitative. All were involved in drafting and/or revising the article, with Angela taking the lead. All have approved the final version of the paper, and are accountable for all aspects of the work.

FUNDING

This work was supported by The National Institute for Health Research - Health Services and Delivery Research: New Research on Community Hospitals, grant number 12/177/13.

COMPETING INTERESTS

None

DATA SHARING

For peer review only

The quantitative datasets for the study are available from the UK Data Archive: Alcock, Peter and Mohan, John (2013). *Third Sector Research Centre research data collection.* [Data Collection]. Colchester, Essex: Economic and Social Research Council. 10.5255/UKDA-SN-850933

REFERENCES

1 NHS England. *Five Year Forward View* London: HMSO 2014.

2 NHS England. *The NHS Long Term Plan* London: HMSO 2019.

3 Dudman J. 'The NHS would collapse without them': The growing role of volunteers. *The Guardian,* 2018. https://www.theguardian.com/society/2018/jul/04/nhs-collapse-without-them-growing-role-volunteers (accessed 10.07.19)

4 Crisp, N. What would a sustainable health and care system look like? *BMJ 2017; 358:j3895 doi: 10.1136/bmj.j3895*

5 Davidson D, Ellis Paine A, Glasby J, *et al.* Analysis of the profile, characteristics, patient experience and community value of community hospitals: A multimethod study. *Health Service Delivery Research 2019;*7(1).

6 Hall J. From cottage to community hospitals: Watlington Cottage Hospital and its regional context, 1874-2009. *Local Popul Stud* 2012;88(1):33-49.

7 Sutton C. Axminster Hospital 1886-1986. 1986.

8 Emrys Roberts M. Cottage Hospitals 1859-1990: Arrival, survival and revival. Dorset: Turn Publications 1991.

9 Cooney E. The Leagues of Hospital Friends. *Public Administration* 1960;38:263-272.

10 Naylor C, Mundle C, Weaks L, Buck D. Volunteering in health and care: Securing a sustainable future. London: King's Fund 2013.

11 Mocroft I, Pharoah C. Coming full circle: charitable funds in London's health. London: King's Fund 2001.

12 Lattimer M. The gift of health. London: Directory of Social Change 1996.

13 Galea A, Naylor C, Buck D, Weaks L. Volunteering in acute trusts in England: Understanding the scale and impact. London: The Kings Fund 2013.

14 Mundle C, Naylor C, Buck D. Volunteering in health and care in England. London: The Kings Fund 2012.

15 Creswell J, Creswell, D. *Research design: Qualitative, quantitative and mixed methods approaches.* London: Sage 2018.

16 O'Cathain A, Murphy E, Nicholl J. The quality of mixed methods studies in health services research. *J Health Serv Res Policy* 2008;13(2)92-98. doi: 10.1258/jhsrp.2007.007074.

17 Halaby C. Panel models in sociological research. *Annual Review of Sociology* 2004;30: 507-544. doi.org/10.1146/annurev.soc.30.012703.110629

18 O'Brien B, Harris I, Beckman T et al. Standards for reporting qualitative research: A synthesis of recommendations. *Acad Med*. 2014;89(9):1245-1251. doi: 10.1097/ACM.0000000000000388.

19 Braun V, Clarke V. Using thematic analysis in psychology. *Qual Res Psychol* 2006;3:77-101. doi:10.1191/1478088706qp063oa.

20 Maguire M. and Delahunt B. Doing a thematic analysis: A practical, step-by-step guide for learning and teaching scholars. *AISHE-J* 2017;8(3): 3351-33514.

21 Hotchkiss R, Unruh L, Fottler M. The role, measurement and impact of volunteerism in hospitals. *Nonprofit and Voluntary Sector Management* 2014;43(6):1111-1128. doi/10.1177/0899764014549057

22 Kang R, Hasnain-Wynia R, Hood M. Hospital commitment to community orientation with its association with quality of care and patient experience. *J Healthc Manag* 2013;58(4): 277-288.

23 Clifford D. Charitable organisations, the great recession and the age of austerity: Longitudinal evidence for England and Wales. *Journal of Social Policy* 2017;46(1): 1-30. doi: 10.1017/S0047279416000325

24 Staetsky L, Mohan, J. *Individual voluntary participation in the United Kingdom: An overview of survey information,* TSRC Working Paper No. 6, Southampton: Third Sector Research Centre 2011.

25 Munoz S-A, Farmer J, Warburton J, Hall J. Involving older people in service co-production: is there an untapped pool of potential participants? *J Rural Stud* 2014;34(3):212-222. doi: 10.1016/j.jrurstud.2014.02.001

26 Mohan J, Bulloch C. *Is there a British 'civic core'? Evidence from the Citizenship Survey.* TSRC Working Paper No. 73; Birmingham: Third Sector Research Centre 2012.

27 Wilson J, Musick M. The Contribution of Social Resources to Volunteering. *Soc Sci Q* 1998;79(4):799–814.

LIST OF FIGURES

Figure 1: League of Friends income distribution in 2014

Figure 2: Community Hospital Leagues of Friends' income sources, by year

Figure 3: Leagues of Friends income and expenditure over time

For peer review only

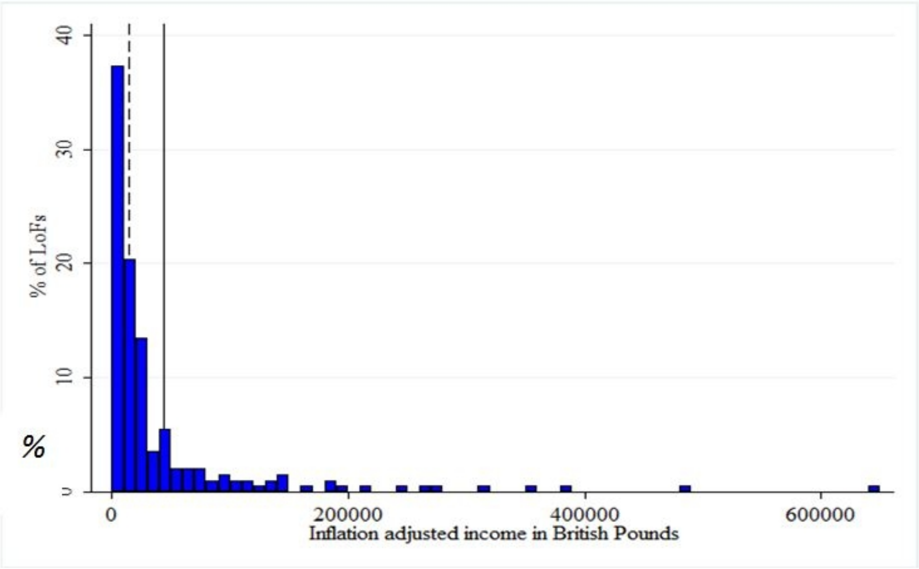

Figure 1: League of Friends income distribution in 2014

Notes: solid line= mean (£45,387); dashed line= median £15,632)

Figure 1: League of Friends income distribution in 2014

168x121mm (120 x 120 DPI)

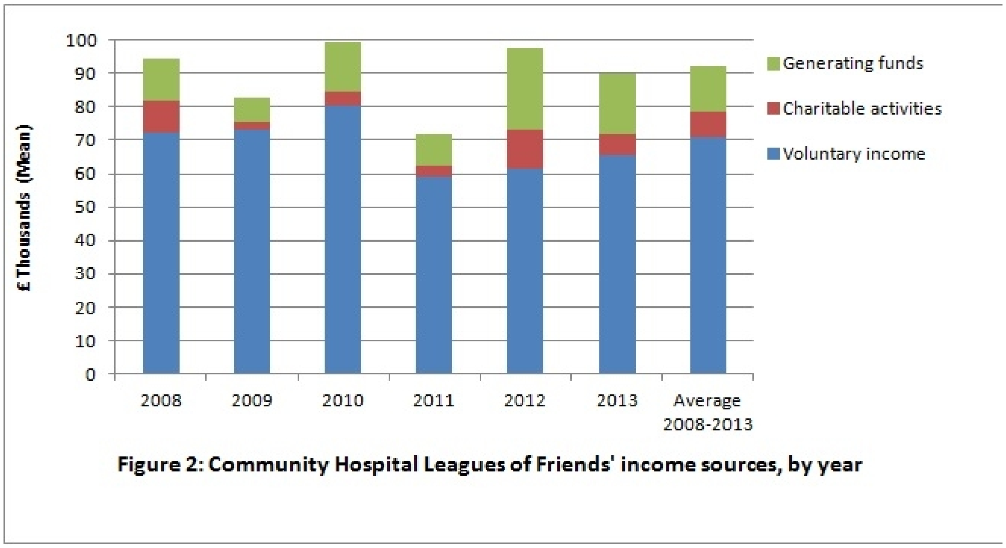

Figure 2: Community Hospital League of Friends' income sources by year

139x75mm (120 x 120 DPI)

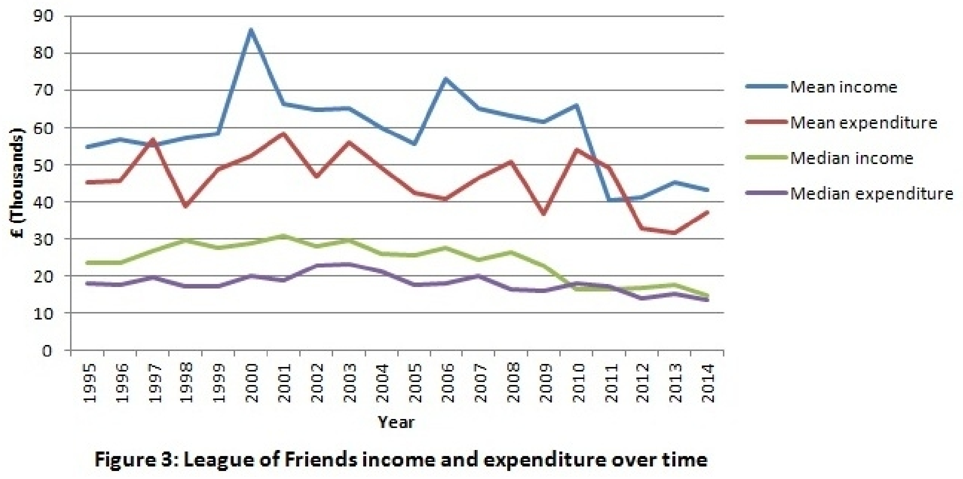

Figure 3: League of Friends income and expenditure over time

237x126mm (72 x 72 DPI)

Communities as 'renewable energy' for health care services? A multi-methods study into the form, scale, and role of voluntary support for community hospitals in England

Angela Ellis Paine, Daiga Kamerāde, John Mohan and Deborah Davidson

Supplementary file: Interview topic guide summaries
Community Hospital research study: Profile, patient and carer experience, and community involvement and value

Staff

1) About you
   - Could you briefly tell me about your role in this community hospital?
   - How and why did you come to work at this hospital?
   - What is it like as a place to work?
2) Organisational background
   - Can you give me a basic introduction to this Community Hospital? (prompt for *history, services, staff, volunteers, funding, challenges and opportunities)*
3) Community context
   - Can you tell me a little bit about the local community: what is it like to live/work here?
4) Patient experience
   - How would you describe the patient experience at this community hospital?
   - Tell me about the main groups of patients who use this hospital?
   - What factors do you think are most significant in shaping patient experience?
   - What do you think of the facilities at this hospital? What kind of feedback do you get from the patients/carers about the facilities here?
   - What are the relationships like between patients and staff here?
   - What do you think about the level of information the hospitals provide the patients regarding their care, hospital facilities and wider services?
   - How involved do you think patients are in decisions about their care? In the hospital?
5) Community involvement
   - What are the different ways in which voluntary groups and the local community are involved in the Hospital, as providers, patients and residents? (probe around volunteering, governance, expert patients, services etc)
   - What does the community contribute to the hospital?
   - How is this organised and managed? (probe around recruitment, support, retention, use of external organisations)
   - How would the hospital be affected if the community did not get involved?
   - Do you think there is any relationship between community involvement and patient experience?
6) Value of community hospital to community
   - What does the community hospital contribute to the local community?
   - How would the community be affected if the community hospital wasn't here?
   - What does this community hospital mean to you?

For peer review only

Community stakeholders:

1) About you
    - Could you briefly tell me about you (and your role as [X])?
    - In what ways are you/your organisation involved in the community hospital?
2) Community hospital
    - How would you describe this community hospital?
3) Community context
    - Can you give me a basic introduction to the local community? What is it like to live/work here?
4) Patient experience
    - How would you describe the patient experience at this community hospital?
    - What factors do you think are most significant in shaping patient experience? (e.g. facilities, relationships, information, involvement)
5) Community involvement
    - What are the different ways in which voluntary groups and the local community are involved in the Hospital, as providers, patients and residents? (probe around volunteering, governance, expert patients, services etc)
    - What does the community contribute to the hospital?
    - How is this organised and managed? (probe around recruitment, support, retention, use of external organisations)
    - How would the hospital be affected if the community did not get involved?
    - Is there a connection between community involvement and patient experience?
6) Value of community hospital to community
    - What does the community hospital contribute to the local community?
    - How would the community be affected if the community hospital wasn't here?
    - What does the community hospital mean to you?

Volunteers

1) About you
    - Can you tell us a little bit about yourself, and about your role as a volunteer at [this] community hospital?
2) Getting involved
    - How did you come to be involved as a volunteer with the hospital? (prompt around being involved through another organisation, receiving services, or connections through family etc)
    - What motivated you to get involved?
3) Volunteering role
    - Can you tell us a bit more about what your role as a volunteer here? What does a typical day as a CH volunteer involve?
    - Has your role evolved over the course of the involvement in the CH?
    - Have you been able to do the kind of volunteer-roles that you hoped? If not, why not?
    - How frequently do you volunteer for the CH?
4) Volunteer support
    - What kind of support have you received as a volunteer at the hospital? (prompt for induction, training, supervision)

For peer review only

- How would you describe the relationship between volunteers and paid staff at the hospital?
- Overall, how helpful do you feel the support for volunteers is at the hospital? What is particularly good? What could be improved?

5) Volunteering outcomes
- What difference do volunteers make to the community hospital as a whole?
- What impact do volunteers have on the staff who work here?
- What difference does the role of volunteers make to hospital patients, and carers? Can you give me any specific examples?
- Is there anything that could be done differently to strengthen the outcomes for service users?
- What difference has being involved as a volunteer at the CH made to you? (prompt for skills; friendships; enjoyment; employability)
- Is there anything you don't like about being a volunteer here?

6) Value of community hospital to community
- What does the community hospital contribute to the local community?
- How would the community be affected if the community hospital wasn't here?
- What does this community hospital mean to you?

Carer:

1) About you
- Could you briefly tell me about you and your relationship to [patient]?

2) Services
    - What are the main services that you have had experience of through being a carer for a patient at this hospital?

3) Carer experience
- Can you tell us about your experience of being a carer of a patient at [X] community hospital? (prompt for: care, support, treatment, facilities, relationships, involvement, feelings)
- Was there anything that was particularly good about being a carer of a patient here?
- Was there anything that was particularly difficult about being a carer of a patient here?
- Was there anything that could have been done better to improve your experience of being a carer of a patient at [x] Community Hospital?

4) Community involvement
- Have you been involved in the CH in any other way, such as being a patient, working here or being a volunteer?
- How involved do you think the local community is in the hospital?
- How would the hospital be affected if the community did not get involved?

5) Value of community hospital to community
- What does the community hospital contribute to the local community?
- How would the community be affected if the community hospital wasn't here?
- What does the community hospital mean to you?

For peer review only

Patients (discovery interview)

Part A

1) We would like to hear about your experiences of this [name] community hospital. You may find it helpful to look at this picture and think about different elements of your experiences and factors that may have shaped them.

Prompts, if not covered in story, for: facilities, relationships, involvement in decisions, feelings

Part B

1) Was there anything that was particularly good about your experience of being a patient at [X] Community Hospital?
2) Was there anything that wasn't particularly good about your experience of being a patient at [x] Community Hospital?
3) What would have improved your experience of being a patient at [x] Community Hospital?
4) Have you ever been involved in the hospital in any way other than as a patient, such as being a member of staff, or a volunteer?
5) Finally a question we are asking everyone we speak with is "What does the Community Hospital mean to you?"

For peer review only

Reporting checklist for qualitative study.

Based on the SRQR guidelines.

Instructions to authors

Complete this checklist by entering the page numbers from your manuscript where readers will find each of the items listed below.

Your article may not currently address all the items on the checklist. Please modify your text to include the missing information. If you are certain that an item does not apply, please write "n/a" and provide a short explanation.

Upload your completed checklist as an extra file when you submit to a journal.

In your methods section, say that you used the SRQRreporting guidelines, and cite them as:

O'Brien BC, Harris IB, Beckman TJ, Reed DA, Cook DA. Standards for reporting qualitative research: a synthesis of recommendations. Acad Med. 2014;89(9):1245-1251.

		Reporting Item	Page Number
**Title**			
	#1	Concise description of the nature and topic of the study identifying the study as qualitative or indicating the approach (e.g. ethnography, grounded theory) or data collection methods (e.g. interview, focus group) is recommended	1&2
**Abstract**			

For peer review only

	#2	Summary of the key elements of the study using the abstract format of the intended publication; typically includes background, purpose, methods, results and conclusions	1&2
**Introduction**			
Problem formulation	#3	Description and signifcance of the problem / phenomenon studied: review of relevant theory and empirical work; problem statement	3-4
Purpose or research question	#4	Purpose of the study and specific objectives or questions	3-5
**Methods**			
Qualitative approach and research paradigm	#5	Qualitative approach (e.g. ethnography, grounded theory, case study, phenomenolgy, narrative research) and guiding theory if appropriate; identifying the research paradigm (e.g. postpositivist, constructivist / interpretivist) is also recommended; rationale. The rationale should briefly discuss the justification for choosing that theory, approach, method or technique rather than other options available; the assumptions and limitations implicit in those choices and how those choices influence study conclusions and transferability. As appropriate	6-7

For peer review only

		the rationale for several items might be discussed together.	
Researcher characteristics and reflexivity	#6	Researchers' characteristics that may influence the research, including personal attributes, qualifications / experience, relationship with participants, assumptions and / or presuppositions; potential or actual interaction between researchers' characteristics and the research questions, approach, methods, results and / or transferability	7
Context	#7	Setting / site and salient contextual factors; rationale	6-7
Sampling strategy	#8	How and why research participants, documents, or events were selected; criteria for deciding when no further sampling was necessary (e.g. sampling saturation); rationale	7
Ethical issues pertaining to human subjects	#9	Documentation of approval by an appropriate ethics review board and participant consent, or explanation for lack thereof; other confidentiality and data security issues	17
Data collection methods	#10	Types of data collected; details of data collection procedures including (as appropriate) start and stop dates of data collection and analysis, iterative process, triangulation of	5-8

For peer review only

		sources / methods, and modification of procedures in response to evolving study findings; rationale	
Data collection instruments and technologies	#11	Description of instruments (e.g. interview guides, questionnaires) and devices (e.g. audio recorders) used for data collection; if / how the instruments(s) changed over the course of the study	7 (with topics guides as supplementary doc)
Units of study	#12	Number and relevant characteristics of participants, documents, or events included in the study; level of participation (could be reported in results)	7
Data processing	#13	Methods for processing data prior to and during analysis, including transcription, data entry, data management and security, verification of data integrity, data coding, and anonymisation / deidentification of excerpts	7
Data analysis	#14	Process by which inferences, themes, etc. were identified and developed, including the researchers involved in data analysis; usually references a specific paradigm or approach; rationale	7

For peer review only

Techniques to enhance trustworthiness	#15	Techniques to enhance trustworthiness and credibility of data analysis (e.g. member checking, audit trail, triangulation); rationale	7-8

Results/findings

Syntheses and interpretation	#16	Main findings (e.g. interpretations, inferences, and themes); might include development of a theory or model, or integration with prior research or theory	8-15
Links to empirical data	#17	Evidence (e.g. quotes, field notes, text excerpts, photographs) to substantiate analytic findings	8-15

Discussion

Intergration with prior work, implications, transferability and contribution(s) to the field	#18	Short summary of main findings; explanation of how findings and conclusions connect to, support, elaborate on, or challenge conclusions of earlier scholarship; discussion of scope of application / generalizability; identification of unique contributions(s) to scholarship in a discipline or field	15-16
Limitations	#19	Trustworthiness and limitations of findings	8, 16

Other

For peer review only

Conflicts of interest	#20	Potential sources of influence of perceived influence on study conduct and conclusions; how these were managed	17
Funding	#21	Sources of funding and other support; role of funders in data collection, interpretation and reporting	17

None The SRQR checklist is distributed with permission of Wolters Kluwer © 2014 by the Association of American Medical Colleges. This checklist can be completed online using https://www.goodreports.org/, a tool made by the EQUATOR Network in collaboration with Penelope.ai

For peer review only
